# *eClock*: An ensemble-based method to accurately predict ages with a biased distribution from DNA methylation data

**DOI:** 10.1371/journal.pone.0267349

**Published:** 2022-05-06

**Authors:** Yu Liu

**Affiliations:** Laboratory of Pathology, Center for Cancer Research, National Cancer Institute, Bethesda, Maryland, United States of America; University of Bonn, Institute of Experimental Hematology and Transfusion Medicine, GERMANY

## Abstract

DNA methylation is closely related to senescence, so it has been used to develop statistical models, called clock models, to predict chronological ages accurately. However, because the training data always have a biased age distribution, the model performance becomes weak for the samples with a small age distribution density. To solve this problem, we developed the R package *eClock*, which uses a bagging-SMOTE method to adjust the biased distribution and predict age with an ensemble model. Moreover, it also provides a bootstrapped model based on bagging only and a traditional clock model. The performance on three datasets showed that the bagging-SMOTE model significantly improved rare sample age prediction. In addition to model construction, the package also provides other functions such as data visualization and methylation feature conversion to facilitate the research in relevant areas.

## Introduction

DNA methylation is a heritable epigenetic modification with an essential role in various physiological and pathological processes [1–4]. Its most common form is the covalent attachment of a methyl group to the 5-carbon atom of DNA cytosine, which usually generates a 5‑methylcytosine (5mC) in the context of cytosine–guanine dinucleotides (CpGs) [5].

DNA methylation and senescence always have a close relation [6, 7]. Hence, some statistical models, called clock models, have been developed to predict chronological ages from DNA methylation sites [8–11]. The most important one is a clock model using 353 CpG sites to predict ages on various human tissues, giving a high correlation between the actual ages and the predicted DNA methylation (DNAm) ages [8]. Further studies show that DNAm ages are also associated with diseases, such as cancer and cardiovascular disease [12–14], pointing to the utility of DNAm age as a biomarker of biological aging [15].

In addition to lifespan age, there are also clock models used to predict gestational age, mainly based on the DNA methylation status of the placenta [16, 17]. The predicted DNAm gestational age also highly correlates with the chronological one and shows a close relationship with some pregnancy complications, such as preeclampsia with accelerated placental DNAm aging [16].

A clock model is a penalized regression model using the squared error loss function to calculate empirical risk and the norm of the regression coefficients to calculate structural risk. The model performance heavily depends on the data quality, and many factors can influence this aspect. In addition to the experimental techniques to build the Infinium DNA methylation microarray, which are critical to the data quality of a single sample, the age distribution of the whole training sample set also plays an important role. A biased distribution can make a model perform poorly in predicting ages with a low distribution density.

For instance, when training a gestational age clock model from placental methylation, a sample can only be collected after delivery of the baby and the placenta. So most samples have a gestational age greater than 30 weeks, which corresponds to moderate preterm and full-term births. For samples with a further younger gestational age, they are scarce, which makes the sample distribution seriously biased to large gestational ages and impairs the ability of the trained model to predict small ones. However, differences in gestational age as small as one week can significantly influence neonatal morbidity and mortality and long-term outcomes [18–23]. Hence, the model’s accuracy across the whole gestational age range becomes essential.

To solve this problem, we developed the R package *eClock* (ensemble-based clock). It improves the traditional machine learning strategy in handling the imbalance problem of category data [24], and combines bagging and SMOTE (Synthetic Minority Over-sampling Technique) methods to adjust the biased age distribution and predict DNAm age with an ensemble model. This is the first time applying these techniques to the clock model, bringing a new framework for clock model construction. *eClock* also provides other functions, such as training the traditional clock model, displaying features, and converting methylation probe/gene/DMR (DNA methylation region) values. To test the performance of the package, we used 3 different datasets, and the results show that the package can effectively improve the clock model performance on rare samples.

## Materials and methods

### Package overview

**Fig 1:**
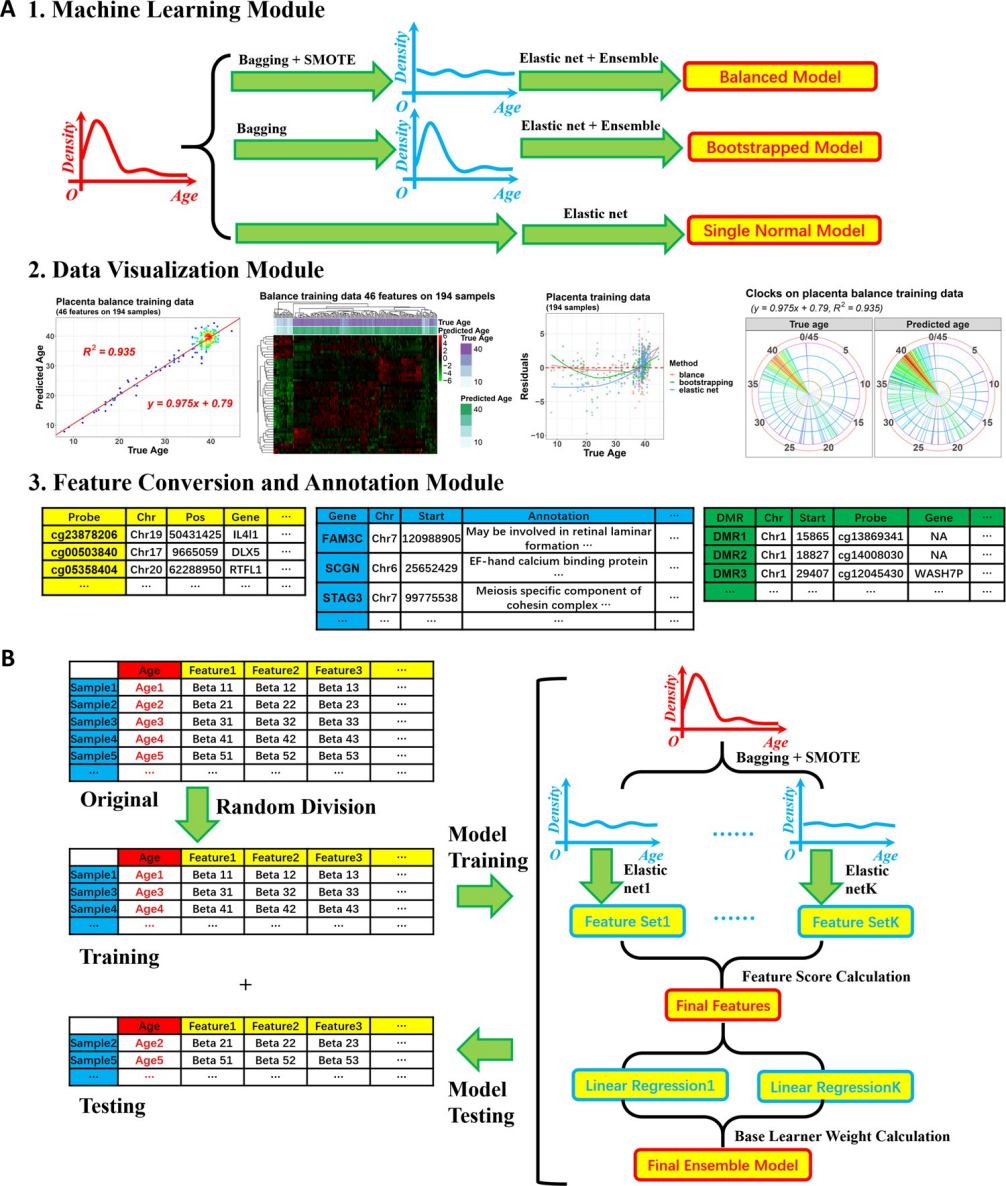
Package workflow. (A) The package has three modules: the machine learning module, the data visualization module, and the feature conversion and annotation module. (B) Workflow of the balanced clock model with several steps on data distribution adjustment (bagging coupled with SMOTE), elastic net base learner training, selected feature integration, linear regression base leaner training, and base learner ensemble.

The package has three modules (Fig 1A). The first one is a machine learning module for clock model construction. For datasets with a biased age distribution, after training/testing sets division, it adjusts the distribution on the training set using a combination of bagging and SMOTE methods and generates clock models in an ensemble form. Then, the testing set without age adjustment is used to evaluate the model performance. For datasets without age bias, normal clock models using the single penalized regression method or bagging ensemble models without age distribution adjustment can be built. The second module is a data visualization module. It carries on the results from the first module and makes various plots to show the model performance and the features selected. The third module is for Infinium DNA methylation microarray data. It converts the DNA methylation value from probe level to the gene or DMR level so that the candidate features for training a clock model can be probes and genes, and DMRs. To explain the biological significance of these features, the package also automatically annotates the methylation probes, genes, or DMRs selected.

### Data collection and preprocessing

The Infinium 27K and 450K BeadChip data on normal and preeclampsia human placentas covering various gestational ages (from 8wk to 45wk), were obtained from Gene Expression Omnibus (GEO) datasets GSE31781 [25], GSE36829, GSE59274 [26], GSE74738 [27], GSE69502 [28], GSE98224 [29, 30], GSE125605, GSE100197, GSE75196, and GSE73375 (Table 1). Among them, GSE98224, GSE125605, GSE100197, GSE75196, and GSE73375 contained both normal and preeclampsia placental data. *SeSAMe* was used to perform data preprocessing [31, 32]. Then, we merged the datasets so that only their overlapping probes shared by the Illumina 27K and 450K platforms were kept.

**Table 1:** Dataset information.

Dataset	Sample origin	Platform	Sample Size	Age/Gestational Age
GSE31781	Placenta	27K	30 normal	8-42wk
GSE36829	Placenta	27K	48 normal	37-42wk
GSE59274	Placenta	27K	23 normal	28-41wk
GSE74738	Placenta	450K	28 normal	36-42wk
GSE69502	Placental chorionic villi	450K	16 normal	14-24wk
GSE98224	Placenta	450K	18 normal + 30 preeclampsia	27-40wk
GSE125605	Placenta	450K	19 normal + 22 preeclampsia	38-45wk
GSE100197	Placenta	450K	43 normal + 22 preeclampsia	25-40wk
GSE75196	Placenta	450K	16 normal + 8 preeclampsia	32-40wk
GSE73375	Placenta	450K	17 normal + 19 preeclampsia	22-40wk
GSE41037	Whole blood	27K	394 normal + 325 schizophrenia	16-88y
GSE41826	Post mortem frontal cortex	450K	145 neron, glia, and bulk samples from 29 normal + 29 depression	13-79y

We downloaded the Illumina 27K dataset on whole blood samples from GSE41037 [7]. It contained 394 healthy samples and 325 schizophrenia patient samples and covered the lifespan age from 16 to 88 years old. Because schizophrenia status had a negligible effect on age relationships [7, 8], we ignored it in this analysis.

We downloaded the Illumina 450K dataset on post mortem frontal cortex from GSE41826 [33]. It included 145 neuron, glia, and bulk samples from 29 normal and 29 depression donors and covered the lifespan age from 13 to 79 years old. Because no evidence showing the disease status accelerated aging [8], we ignored it during the analysis.

### Age distribution adjustment

The function *resamplebin* in the package adjusted a biased age distribution to a balanced one. It equally divided the original age range of the training dataset into various bins. For each bin, under or over-sampling was performed on the samples until the final sample number reached the ceiling of (total training sample number/bin number). Hence, if a bin contained too many samples, it would be under-sampled with bootstrapping, while if a bin only had a few samples, it would be over-sampled. However, this over-sampling was not conducted with bootstrapping because resampling on only a few samples had a risk of over-fitting. Instead, the function *resamplebin* used a modified SMOTE method to synthesize new samples via interpolation. If a sample were randomly selected, *resamplebin* would search for its nearest neighbor within the bin and calculate the vector recording their feature value differences. After that, a random number between -0.5 and 0.5 would be generated to multiply this vector. Then, the new vector would be added to the original sample vector. The result was the synthesized sample, and its age was assigned as that of the original sample.

On the other hand, to rescue the samples discarded from the bins undergoing under-sampling, bagging was used on the whole training set to construct an ensemble model. Hence, although each base learner in it lost some sample information due to the under-sampling, for the whole ensemble, all the samples were used.

As to the bin width, its initialized value was 1 week for gestational age and 1 year for lifespan age, but if any of the bins contained less than 2 original samples, the bin width would be increased by 1 week or 1 year until each bin had at least 2 samples. Compared with the original age distributions, the adjusted ones were more balanced.

In addition to *resamplebin*, we also offered another function, *simpleboot*, which could do normal bootstrapping on the training dataset without adjusting its distribution to build a normal bagging-based ensemble model.

### Clock model construction

The function *singlebalance* trained the balanced clock models. It conducted several steps (Fig 1B). First, it randomly divided the whole input dataset into training and testing ones.

Then, *resamplebin* was called several times for the training dataset to generate several subsets from it with balanced age distribution. Each subset would be used to train a base learner fitting the sample ages with the methylation features via elastic net regularization, $\underset{(\beta_{0},\beta )\in R^{p+1}}{\min}\frac{1}{N}\sum_{i=1}^{N}l(y_{i},\beta_{0}+\beta^{T}x_{i})+\lambda [(1-\alpha )\;\frac{1}{2}∥\beta {∥}_{l2}^{2}+\alpha ∥\beta {∥}_{l1}]$. The function *singlebalance* performed this in virtue of the R package *glmnet* [34–36]. In the formula above, the first part was the squared error loss function calculating the empirical risk, while the second part was the elastic net penalty. The *α* parameter there controlling the balance of L1 and L2 penalties was set using the parameter *alphas* of the function *singlebalance* (set as 0.5 here). At the same time, the regularization constant *λ* was chosen during 10-fold cross-validation. After that, *singlebalance* chose the *α*−*λ* combination giving a cross-validation error within one standard error of the minimum to construct the elastic net model.

Each base learner used this elastic net method to select a set of features. Then the features from all the base learners were combined, and the ones with top feature scores were selected for the following calculation. The function *singlebalance* calculated the feature scores referring to a method on category data [24], and using the formula, $(F_{p}^{+}-F_{p}^{-})*\bar{\beta_{p}}$, where $F_{p}^{+}=\frac{1}{K}\sum_{k=1}^{K}I(\beta_{p}^{\left(k\right)}>0),\;F_{p}^{-}=\frac{1}{K}\sum_{k=1}^{K}I(\beta_{p}^{\left(k\right)}<0)$ represented the percentages of base learners with a coefficient larger or smaller than 0 for the *p*th feature, and $\bar{\beta_{p}}=\frac{1}{K}\sum_{k=1}^{K}\beta_{p}^{\left(k\right)}$ was the averaged coefficient value of this feature across all the *K* base learners.

Then, these selected features were given back to each sample subset to generate a linear regression model and predict sample ages using the feature beta values. To ensemble these linear base learners, each of them was assigned a weight, which was calculated according to the regression R square between the true sample age and base learner predicted age on the whole training dataset. Only the base learners with an R square greater than 0.5 would be kept to do the final ensemble, and their weights would be $0.5*\log\left(\frac{R^{2}}{1-R^{2}}\right)$ with the following scale to the sum of 1. The prediction result of the whole ensemble model was the weighted sum of the base learner results.

If the parameter *balancing* were FALSE, *singlebalance* would not make distribution adjustment and instead train a normal bagging model using simple bootstrapping to generate subsets for the base learners. Hence, it was also used to build the bootstrapped models.

Another function, *singleselection* in the package, was used to train the normal clock models with a single penalized regression.

The function *crosstraining* conducted the 10-round training/testing division and the model training. For each round, *crosstraining* divided the input data into training and testing randomly and trained a balanced, or bootstrapped, or normal clock model via calling the function *singlebalance* or *singleselection*.

### Model evaluation and data visualization

The scatter plots, residual plots, and clock plots were generated using several functions: *scatterplot*, *residualplot*, *residualcomp*, and *clockplot*. These functions were also integrated into *singlebalance*, *singleselection*, and *crosstraining* so that they could also generate the plots.

### Methylation probe annotation

The function *extractprobes* in the package extracted the selected probes from the model results. Then the probes were transferred to the function *probeannotation* to get their annotation information.

### Biological function enrichment analyses

The function enrichment results for the feature-relevant genes were generated using the R package *EnirchR* [37, 38].

### Preeclampsia sample DNAm gestational age calculation

The function *ensemblepredict* could predict response values for new data using a trained elastic net or ensemble model. So the preeclampsia DNA methylation data and the model trained from the normal samples were transferred to *ensemblepredict* together. The model prediction values were the DNAm gestational ages of the preeclampsia samples.

### Probe value summarization to gene

The function *probetogene* in the package could summarize the probe beta values to corresponding genes. For a gene, the beta values of probes in its TSS200, TSS1500, and 1stExon regions were averaged to get the gene beta value. The regions could be tuned by the parameter *group450k850k*, which could choose one or more gene regions from “TSS200”, “TSS1500”, “1stExon”, “5’UTR”, “3’UTR”, and “Body”. Only the probes within the gene regions chosen by this parameter would be used to calculate the gene beta values.

### Gene annotation

The function *extractprobes* extracted the selected genes from the models with genes as features. Then these genes were transferred to the function *geneannotation* to annotate their genomic coordinates and functions.

### Probe value summarization to DMR and DMR annotation

The function *probetodmr* summarized the probe beta values to DMRs. First, it divided the probes into different clusters according to their genomic coordinates. If the genomic distance between 2 probes were less than 500 bp, they would be grouped into the same cluster, and each cluster was considered a DMR. Then, for each DMR, the beta values of the probes within it would be averaged and used as the DMR value. In addition, *probetodmr* also annotated the DMRs. It reported their positions, probes, and the genes related to each DMR.

### Balance index calculation

The function *resamplebin* calculated the balance index for a dataset, with its parameter *balanceidx* set as TRUE. First, the kernel density of the response was computed on 512 equally spaced points across the range of the response. Then, the variance of these density values around their mean was calculated, and the minus log10 value of the variance was the final balance index. A small balance index indicated a biased response distribution, while a larger one meant a more balanced distribution.

## Results

### The balanced clock model has an advantage in fitting rare samples with a young gestational age

We first used the package on a gestational age dataset from various studies on placental DNA methylation (Table 1). After data preprocessing and combination, we kept the probes with high data quality and shared by the Illumina 27K and 450K platforms. The final dataset contained 258 normal (or uncomplicated) placenta and 101 preeclampsia samples, with 18626 probes. Among the normal samples, 3/4 of them were randomly selected as training samples (194 samples) to train the clock models. The remaining 64 samples without any participation in the training process were used as a testing dataset to check the model performance on normal samples. The 101 preeclampsia samples were used to check the model performance on disease samples.

**Fig 2:**
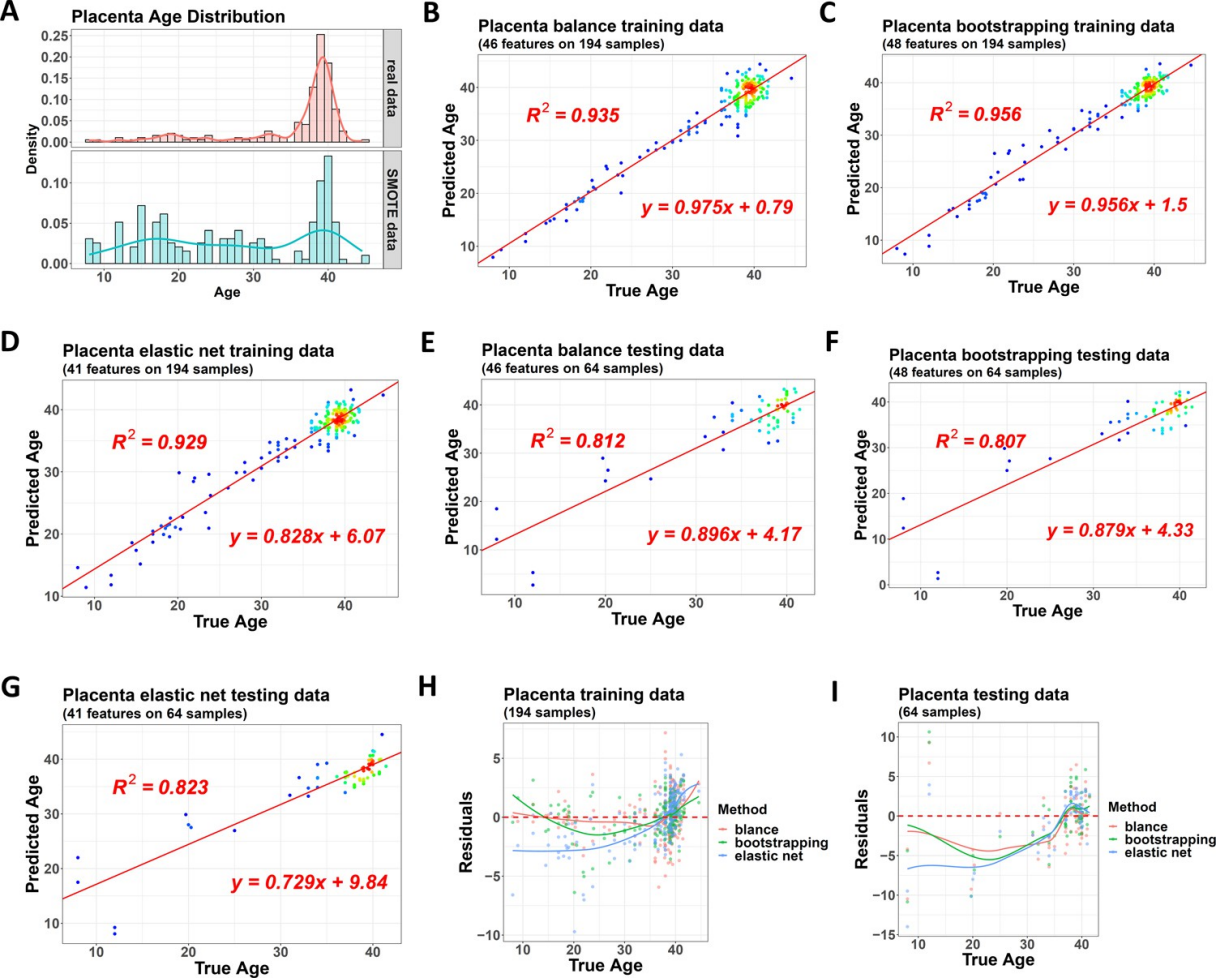
Performance of the three clock models on the gestational age dataset. (A) The original training dataset has a biased gestational age distribution (upper). At the same time, the balanced clock model adjusts it to a balanced one (lower), with samples from all the 10 base learners combined and shown here, including synthesized samples from SMOTE and repeated samples due to bootstrapping and bagging. (B) to (D) Performance of the balanced model (B), bootstrapped model (C), and single normal model (D) on the same training dataset. The color gradients of the dots indicate the density of the samples. (E) to (G) Performance of the three models on the same testing dataset. (H) and (I) Residuals of the three models in training and testing datasets.

The gestational age of the training dataset showed a seriously biased distribution, with most samples concentrated between 36 weeks and 42 weeks, while rare samples had a gestational age smaller than that (Fig 2A). To avoid its negative effect on the model performance, we used the package to adjust this distribution on the training dataset first and then constructed a balanced clock model with the bagging-SMOTE strategy, as described in the Materials and methods section (Fig 2A). Meanwhile, we also trained another two clock models. The first was a single panelized regression model (normal model). The second was a bagging-based ensemble model (bootstrapped model), but it did not make any distribution adjustment. Both the balanced model and the bootstrapped one contained 10 base learners. We then compared the model performance on the original training and testing datasets without any age distribution adjustment.

For the same training dataset, the balanced model had an R square of 0.935, the bootstrapped model had an R square of 0.956, and the normal model had an R square of 0.929 (Figs 2B to 2D and S1A to S1C). For the testing dataset, their values were 0.812, 0.807, and 0.823 (Figs 2E to 2G and S1D to S1F). Hence, these models showed a similar performance, and the normal model was a little better given its best R square on the testing set.

However, if checked details, the influence of the biased age distribution became clear. We used the package to generate a residual plot and compared the sample residuals of the three models (Fig 2H and 2I). For the samples with a gestational age greater than 35 weeks, which had a large distribution density, their residuals were similar among the three models. However, for the much broader gestational age range from 8 weeks to 35 weeks, but with a small sample number, the balanced model fitted much better than the other two.

**Fig 3:**
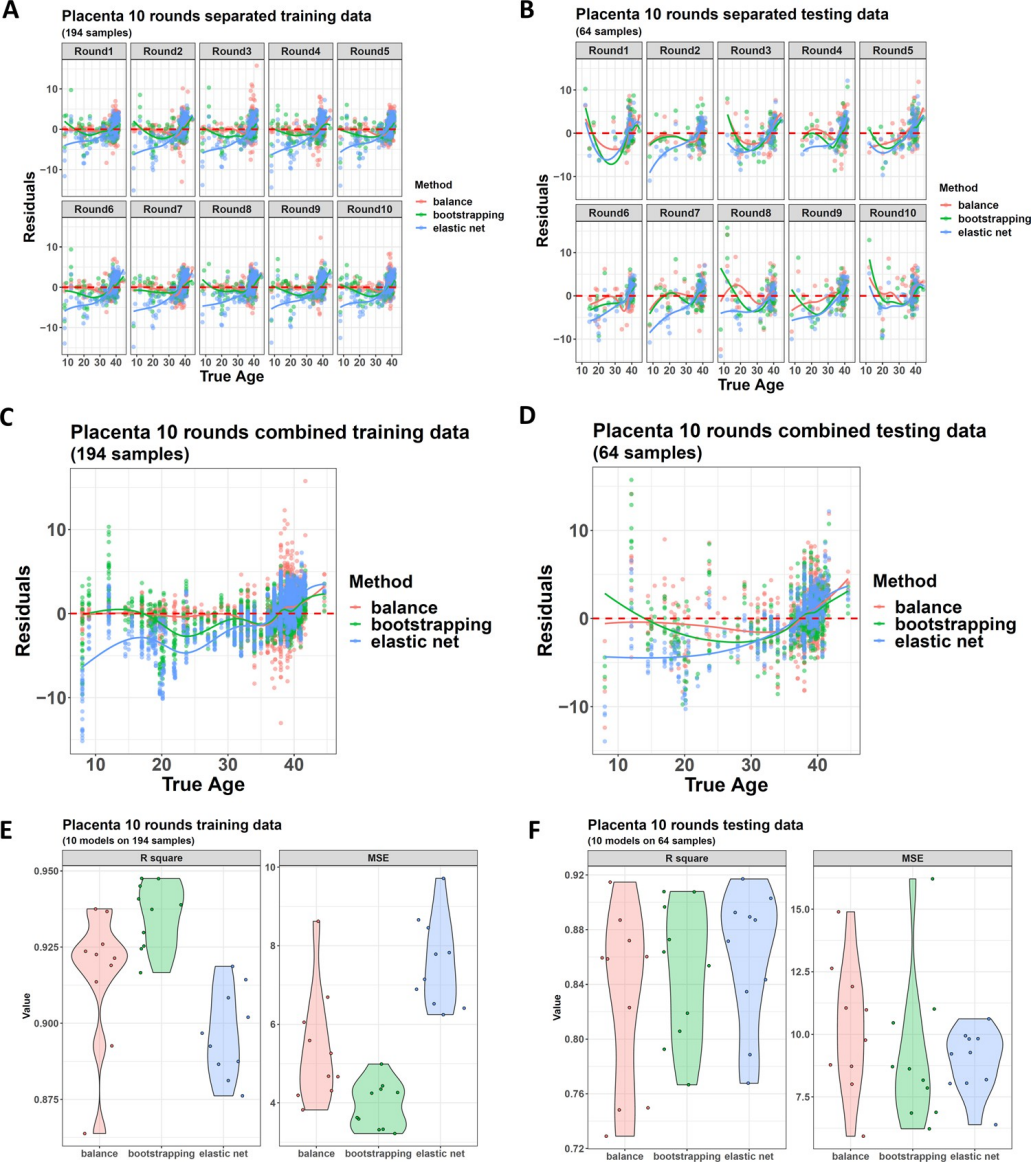
Performance of the three clock algorithms on a 10-round gestational age model construction process. (A) and (B) Residual plots for the 10 new training and 10 new testing datasets generated from the 10 rounds. (C) and (D) Averaged residual curves after combining all the residuals in the 10 rounds for training and testing datasets, respectively. (E) and (F) R squares and MSEs (mean squared errors) for the models generated in the 10 rounds.

Then, we used the package to perform a 10-round model training to get more statistical significance. For each round, the normal samples were re-divided into a new training and a new testing dataset. Three new models were rebuilt from the training without any participation by the testing. The residual plots indicated that the advantage of the balanced model was repeatable across the 10 rounds. For the samples with a rare gestational age, almost all the training and testing datasets showed a better fitting by their balanced models (Fig 3A and 3B). If averaged residuals across the 10 rounds, the final plots also showed the balanced model obtained a residual much closer to 0 (Fig 3C and 3D).

However, if we checked the overall model performance, the three models had a similar performance (Fig 3E and 3F). The advantage disappearance of the balanced model here was because the overall performance was mainly contributed by the samples with a large distribution density, even if their age span was narrow. On these samples, the balanced model did not have a significant advantage.

Hence, this case study concluded that if the main focus was to find a model with good overall performance, all three methods could be considered, and the normal one was a little better. However, if the purpose was to obtain a good performance across the whole age range, the balanced model was the best.

Additionally, we compared the performance of the balanced model with other published gestational age clock models, which were collected in the R package *methylclock* [39]. Among them, the Knight’s model used 148 methylation probes to predict the gestational age, and > 80% of them were covered by the testing dataset here [40]. For other models, because < 80% of their required probes were covered, their performances were not checked. As mentioned above, the balanced model had an R square of 0.812 for the beginning testing dataset. In contrast, for the Knight’s model, its R square was only 3.73e-3 on this testing set (S2A Fig). However, it did not mean the Knight’s model was weak because it was originally trained from cord blood samples rather than placenta samples here. Hence, this weak R square indicated that the models trained from cord blood were unsuitable for the placenta. For MSE, the balanced model had a value of 13.0, while that of the Knight’s model was 336. In addition, Knight’s model also showed huge residuals across all the samples (S2B Fig). The probes used by the Knight’s and the balanced models were largely different. For the 134 probes used by the Knight’s model in this testing set, only 1 was shared with the balanced model (S2C Fig).

### The clock models have a close relation to gestational age and preeclampsia

Next, we checked the DNA methylation probes selected by our 3 models. For the balanced model constructed at the beginning, it selected 46 probes. We used the package to annotate them, and the result showed the probes distributed in regions of CpG Island, N-Shore, S-Shore, N-Shelf, and OpenSea. For the relation to genes, they were located in TSS200, TSS1500, 1stExon, 5’UTR, and GeneBody regions. (S1 Table).

**Fig 4:**
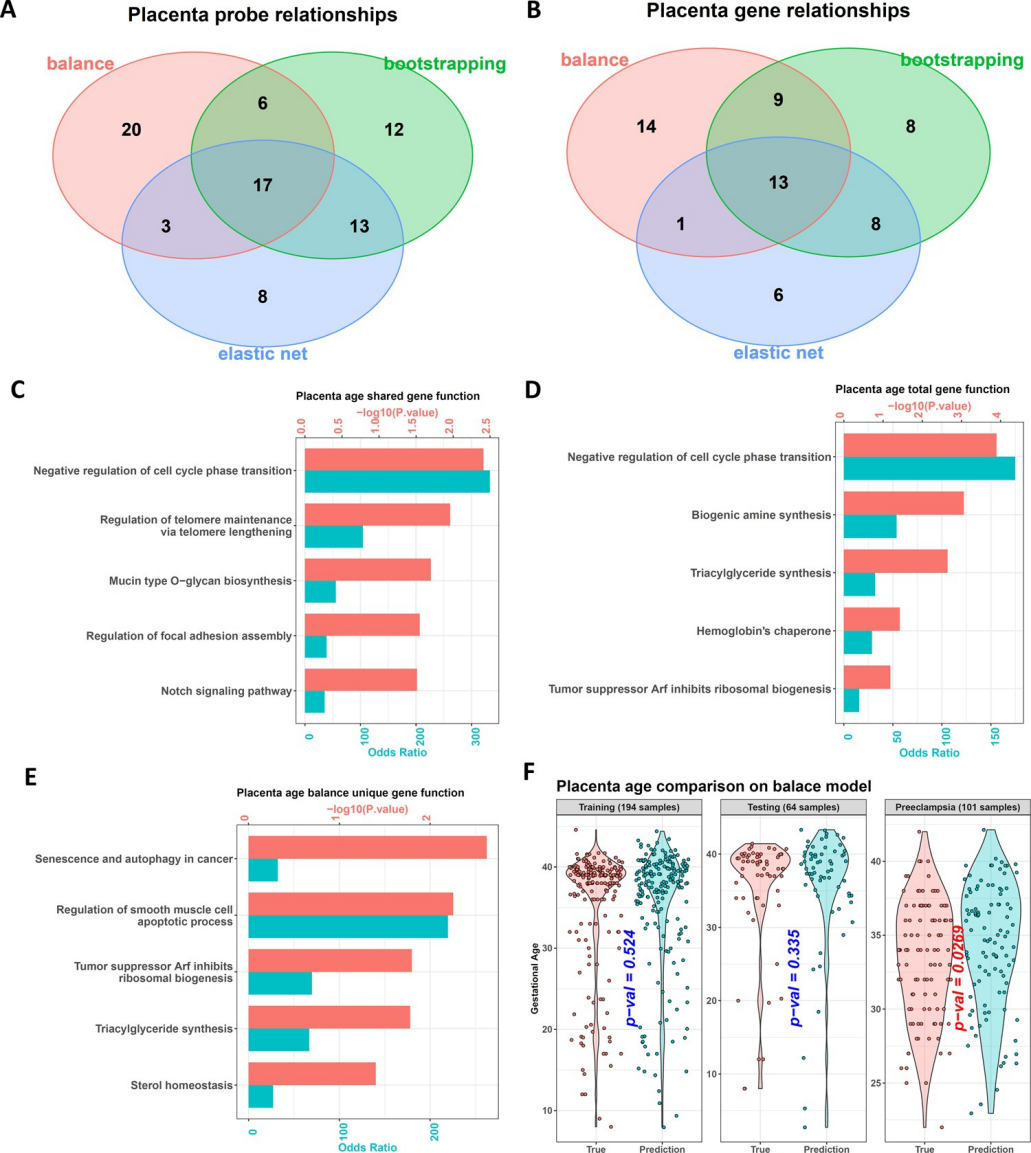
Biological functions of the probes selected from the gestational age dataset. (A) and (B) Venn diagrams showing the relation among the features selected by the three models for probes and genes, respectively. (C) to (E) Biological function enrichment results for the shared genes (C), total genes (D) of the three models, as well as the genes selected by the balanced model uniquely (E). (F) The balanced clock shows that the DNAm gestational ages of normal samples are similar to their chronological ones. However, the DNAm gestational ages of the preeclampsia samples are significantly larger than their chronological ones, indicating the accelerated senescence.

The three models totally selected 79 probes, and 17 were shared by them (Fig 4A). For the genes with TSS200, TSS1500, or 1stExon covered by these probes, totally 59 such genes were found, and 13 were shared (Fig 4B).

For the 13 shared genes, some aging relevant functions were enriched, such as “Regulation of telomere maintenance via telomere lengthening” and "Negative regulation of cell cycle phase transition" (Fig 4C). For the 59 total genes, some synthesis relevant functions were found, including "Biogenic amine synthesis" and “Triacylglyceride synthesis” (Fig 4D). Given the tight connection between senescence and energy consumption [41], these synthetic functions might indirectly influence aging. We also checked the 14 genes uniquely selected by the balanced model. Their functions contained "Senescence and autophagy in cancer" (Fig 4E).

In addition, we searched the GenAge database, a benchmark database of genes related to aging [42], to find the overlapping of all the 59 genes from our models with the genes collected in GenAge. However, we only found 1 overlapped gene (CDKN2A). It was from the balanced model. We then used our package to annotate it, and we found it was a negative regulator of cell proliferation (S4 Table), indicating its methylation level should be positively related to aging. Correspondingly, the sum of its coefficients in the balanced model base learners was also positive (coefficient sum = 12.3).

On the other hand, because differential DNA methylation in the placenta had been shown in preeclampsia [26], a pregnancy complication coupled with accelerated placental aging [16], we next used the models to predict the DNAm gestational age of the 101 preeclampsia samples mentioned above. All three models showed that the DNAm gestational ages of these disease samples were significantly larger than their chronological ones, which was different from the normal samples in the testing dataset. Although these normal samples also did not participate in the model training, their DNAm gestational ages predicted were similar to the chronological ones (Figs 4F and S3A and S3B). These results confirmed the accelerated senescence of preeclampsia placenta [16].

### The balanced clock model has an advantage in fitting whole blood samples with small lifespan age density

We also used the package on a lifespan age dataset from a study on whole blood DNA methylation [7]. It was from Illumina 27K platform and contained 719 samples with lifespan ages from 16 to 88 (Table 1). We used the package to randomly select 3/4 samples into the training dataset (539 samples), and the remaining 180 samples were testing samples.

**Fig 5:**
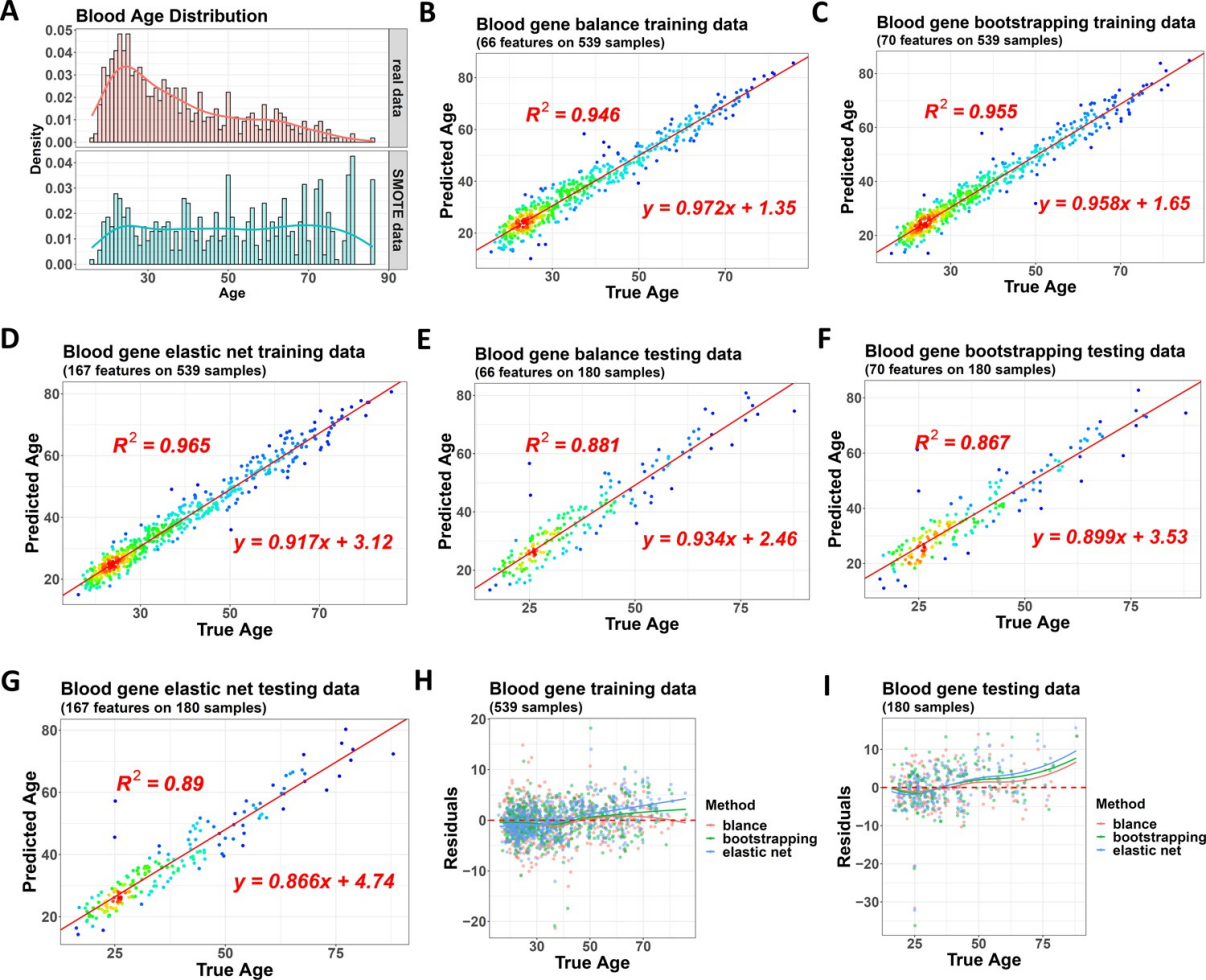
Performance of the three clock models on the whole blood dataset. (A) The original training dataset has a biased lifespan age distribution (upper). At the same time, the balanced clock model adjusts it to a balanced one (lower), with samples from all the 10 base learners combined and shown here, including synthesized samples from SMOTE and repeated samples due to bootstrapping and bagging. (B) to (D) Performance of the balanced model (B), bootstrapped model (C), and single normal model (D) on the same training dataset with genes as features. The color gradients of the dots indicate the density of the samples. (E) to (G) Performance of these three models on the same testing dataset with genes as features. (H) and (I) Residuals of the three models in training and testing datasets.

The three algorithms were used on the probe beta values, and the model performances were evaluated (S4 Fig). In addition, we also converted the original probe beta values to gene beta values via the package and then trained clock models using genes as features. The lifespan age distribution in the training dataset also showed a bias because the samples greater than 40 years old were less than the younger ones and the balanced model adjusted it via the bagging-SMOTE method (Fig 5A).

For the gene-based clocks, the balanced model, bootstrapped model, and single normal model showed similar performance with R squares of 0.946, 0.955, and 0.965 on the training data (Fig 5B to 5D), and 0.881, 0.867, and 0.89 on the testing data (Fig 5E to 5G). However, if looked into the residual status, the balanced model showed better performance than the other two (Fig 5H and 5I).

Hence, the conclusion of this case study was similar to the former one, indicating the normal model had a slight advantage in overall R square, while the balanced model was the best to fit the whole age range.

The features of these models were genes, and the three models selected 179 genes totally and shared 48 of them (S2 Table and S5A Fig). For the total genes, their enriched function included “Oxidative stress induced senescence” (S5B Fig). For the 48 shared genes, "Negative regulation of mitochondrion organization" was noteworthy given the association between senescence and energy metabolism [41] (S5C Fig). We also checked the 92 genes uniquely selected by the single normal model because of its large gene number and found they were enriched in functions such as "snRNA metabolic process" and "oxoacid metabolic process" (S5D Fig).

In addition, we compared the performances of the balanced model (probe-based) with other published lifespan age clock models in the *methylclock* package. For 4 models (Horvath’s model, BNN model, Levine’s model, and Wu’s model), all of them used probes as features, and all of their required probes were covered by the testing dataset here [8, 43–45], and their performances were checked. The result showed that the best model was the BNN model (R square = 0.916, MSE = 20.0), the second-best was our balanced model (R square = 0.901, MSE = 23.7), and the third model was the Horvath’s model (R square = 0.894, MSE = 25.0) (S6A Fig). However, it was noteworthy that the performance of BNN and Horvath’s models should be overestimated because the testing dataset here was a part of their training dataset in their original studies, while for the balanced model, this testing set did not participate in its model training. On the other hand, for the residual distribution, the balanced model still kept its advantage on the low-density samples > 40 years old (S6B Fig). For the required probes of the models, only a few probes were shared by the models (S6C Fig).

Then, for the 179 gene features selected by our gene-based models, we also checked their overlapping with the GenAge database. Only 6 of the 179 genes were recorded by the database. We also mapped the probe features of Horvath’s model and others to genes and then checked their overlapping with GenAge. For Horvath’s model, only 4 genes appeared in the database, while for others, such as Wu’s and Hannum’s models, the number was 2. Hence, this low overlapping was not unique to our models. It indicated that more attention should be paid to exploring the biological significance of the methylation gene features to aging.

For the 6 genes covered by our models and GenAge simultaneously (AGPAT2, E2F1, MAP3K5, TERT, LMNA, and C1QC), E2F1 and C1QC were features selected by all the elastic net, bootstrapped, and balanced models, and the annotation from our package showed that E2F1 was involved in cell cycle and DNA replication. At the same time, C1QC participated in C1 generation (the first component of the serum complement system) (S4 Table). In addition, GenAge showed that the RNA of C1QC was overexpressed in the aging microarray meta-analysis, which was consistent with its negative coefficients in our methylation models (elastic net coefficient = -0.758, bootstrapped base learner coefficient sum = -1.77, balanced base learner coefficient sum = -9.21).

### Distribution adjustment is not suitable for data with an original balanced response

**Fig 6:**
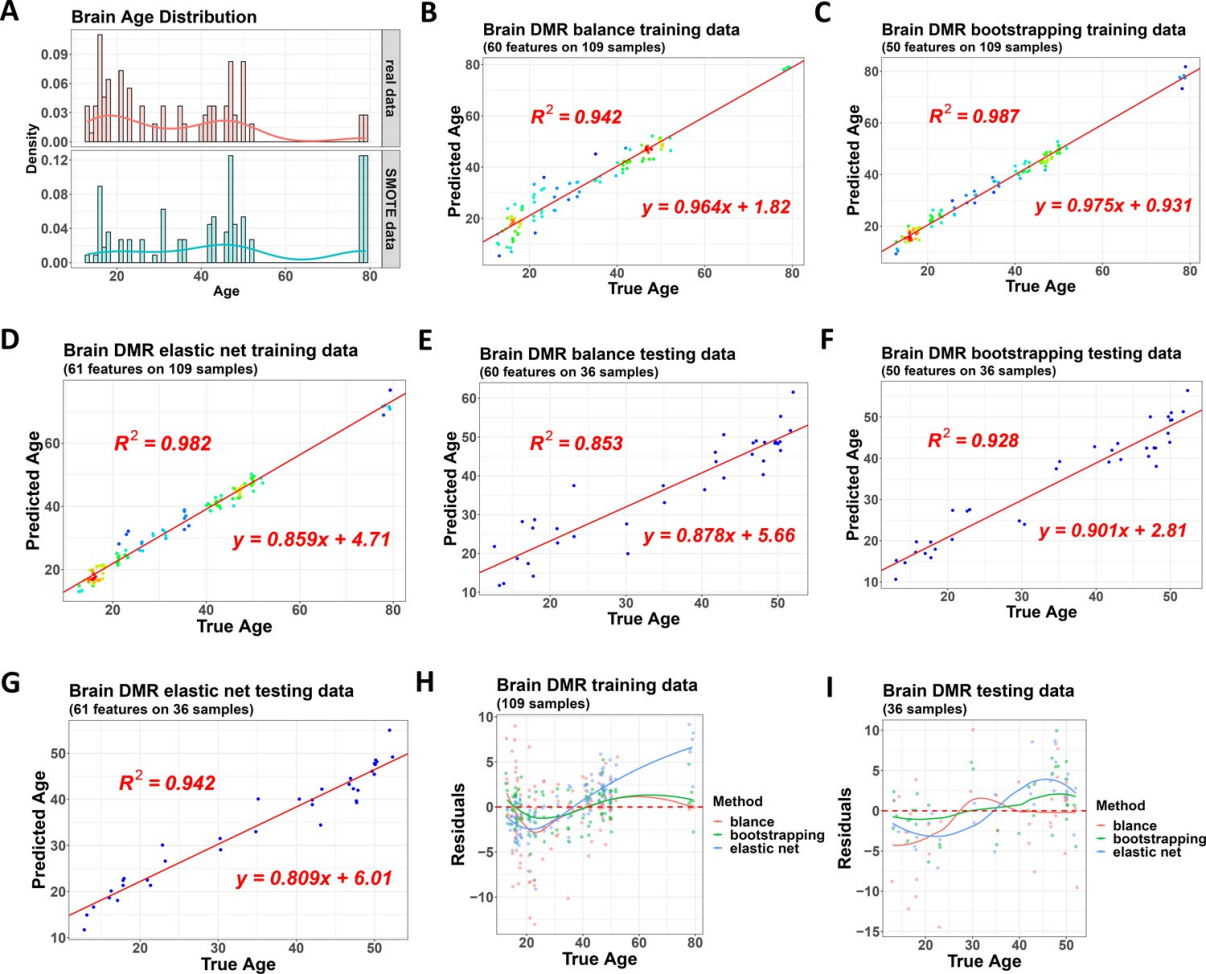
Performance of the three clock models on the 450K brain dataset. (A) The lifespan age distribution of the original training dataset before (upper) and after (lower) the adjustment by the balanced clock model, with samples from all the 10 base learners combined and shown, including synthesized samples from SMOTE and repeated samples due to bootstrapping and bagging. (B) to (D) Performance of the balanced model (B), bootstrapped model (C), and single normal model (D) on the same training dataset with DMRs as features. The color gradients of the dots indicate the density of the samples. (E) to (G) Performance of these three models on the same testing dataset with DMRs as features. (H) and (I) Residuals of the three models in training and testing datasets.

In addition to probes and genes, we also tried to construct clock models using DMRs as features. Hence, we used a 450K-based dataset on 145 samples from the post mortem frontal cortex of the brain [33]. The lifespan age of the samples covered a range from 13 to 79 years old. Initially, it contained 480492 probes, and after the conversion using the package, the probes were clustered to different DMRs, and the whole dataset had 202450 DMRs. The sample age was distributed more uniformly in this dataset than in the previous placenta and whole blood ones, but we still tried the balanced model with distribution adjustment (Fig 6A).

For the three DMR-based clock models, the balanced one had an R square of 0.942 on the training set, while the bootstrapped and the single normal models had values of 0.987 and 0.982 (Fig 6B to 6D). On the testing set, their R squares were 0.853, 0.928, and 0.942 (Fig 6E to 6G). If we checked the residuals, this time, the balanced clock model lost its advantage because, in both training and testing datasets, the bootstrapped model showed a better residual plot (Fig 6H and 6I). This advantage disappearance was also observed in the models using methylation probes as features (S7 Fig).

We attributed this disappearance to the original balanced age distribution in this dataset (Fig 6A), which made the sampling step of the bootstrapped model also generate subsets with a balanced response and then get base learners without performance bias. However, the balanced clock model still used the bagging-SMOTE strategy to generate new response distribution. For this dataset, its original balanced distribution could not be improved significantly, but the synthesized samples from SMOTE introduced more noise, making the performance weaker than the bootstrapped model.

Hence, this case study concluded that the SMOTE-based adjustment should not be made if the age distribution had already been balanced. In this case, the bootstrapped model should be used.

To quantify the imbalance of the original response variable in a dataset, we calculated a statistic named “balance index” using the package. A small balance index indicated a biased distribution, and a larger one indicated a relatively uniform one. For the three datasets used here, the placenta dataset had a balance index of 2.67, the whole blood dataset had a balance index of 4.03, while that of the brain dataset was 4.12, which meant the placenta dataset had the most biased response. In contrast, the brain dataset had the most balanced one. Hence, the balanced model had an advantage in fitting the placenta dataset but performed weaker in the brain dataset, no matter the feature used, methylation probes, or DMRs.

As to DMRs, the three models totally selected 107 of them from the 450K brain dataset and shared 22 of them (S3 Table and S8A Fig). From the DMR annotation result of the package, the total 107 ones covered the TSSs of 42 genes, and 12 were shared by all the models (S8B Fig). The 42 genes were related to translational functions such as “Translation factor activity, RNA binding” and “tRNA processing” (S8C Fig). In contrast, the shared genes exhibited a connection to the neural system by their enriched functions of “Glial cell development”, “Neuroepithelial cell differentiation” and “Regulation of hippo signaling” (S8D Fig).

Furthermore, we compared the performances of the balanced model (probe-based) and the bootstrapped model (probe-based) with other published lifespan age clock models. For 7 models, all of their required probes were covered by the testing dataset, and their performances were checked. For R square, the top 4 models were: Horvath’s model (R square = 0.953), bootstrapped model (R square = 0.918), Hannum’s model (0.897), and balanced model (0.827). For MSE, the ranks of the models changed to bootstrapping model (MSE = 16.4) < Horvath’s model (MSE = 24.3) < balanced model (34.6) < Hannum model (108) (S9A Fig). Hence, Hannum’s model obtained a high R square but a weak MSE, indicating its predicted ages had an overall shift from the true values. For the sample residuals, the bootstrapped and the balanced models showed the largest advantage (S9B Fig). It should be noted that Horvath’s model still had an overestimation problem because the brain testing dataset here was also a part of its training dataset. While for other models, their performances tended to be underestimated because they were originally not trained from brain data [9, 43, 46]. For the required probes of the models, the balanced and the bootstrapped models had no overlapping with others.

We also checked the overlapping between the DMR covered genes and the GenAge database and only found that the bootstrapped model had a gene shared with it (RAE1). The annotation result showed that RAE1 functioned in mitotic bipolar spindle formation and mRNA nucleocytoplasmic transport (S4 Table).

## Discussion

Age-related DNA methylation alteration exists in various species [6, 7, 47, 48]. Hence, DNA methylation has been used to construct clock models to predict chronological age [8–11].

The performance of a clock model is influenced by the age balance, and its accuracy is weakest at the extremes of the age distribution. However, it is usual to meet a dataset with biased age, so it is necessary to deal with this issue. Hence, we developed the R package *eClock*, which includes three kinds of clock models. The first one is a balanced model based on the bagging-SMOTE strategy and can improve the age distribution and train an ensemble model. The second one is a bootstrapped model using bagging but without distribution adjustment. The third is a traditional clock model using a single elastic net regression. We used three datasets to test the package and these three models and found each has its advantages.

The three models have very similar overall R squares, but the single normal model always has a little larger R square on the testing data, so we suggest using this one to optimize the overall R square.

However, to obtain a good performance across the whole age range, the balanced model or the bootstrapped model should be used. The choice between them depends on the original distribution status.

In most cases, this distribution is biased, and the balanced model is more appropriate than the bootstrapped model. Its adjustment step increases the weights of the rare samples, and so the loss function reaches its minimum when the errors are small not only on the major samples but also on the rare ones.

However, for cases that the original data distribution is not biased, such as the brain dataset here, the bootstrapped model is better. Because of the original balance, the bootstrapping step of this model can also get an unbiased distribution for its base learners. Meanwhile, it avoids the noise introduced by the SMOTE step of the balanced model.

To facilitate the judgment on distribution balance and model choice, we introduce the balance index to the package, and a small one indicates a biased distribution. In contrast, a larger one indicates a more balanced status. The balance indexes of the placenta and whole blood datasets are 2.67 and 4.03, and the balanced model shows an advantage. However, for the brain dataset with a balance index of 4.12, the bootstrapped model surpasses the balanced one. Hence, we suggest that if the index is less than 4, the balanced model should be used, and if it is greater than 5, the bootstrapped one should be chosen. If a balance index is between 4 and 5, it is better to try both models.

This package introduces a new framework to clock model construction for the first time and efficiently improves the prediction in rare sample ages. In addition, it also provides other functions such as methylation feature conversion and annotation, which improve the interpretability of the model results. We hope this package can make some contribution to relevant areas.

## Supporting information

S1 Fig Performance of the three models on the gestational age dataset shown by clock plots. (A) to (C) Clock plots generated by our package show the clock model performance on the same training dataset. The scale around the dial indicates the gestational age, and each pointer represents one sample. The color gradients of the pointers indicate the density of the samples. (A) is the result for the balanced model, (B) is for the bootstrapped model, and (C) is for the single normal model. (D) to (F) Performance of these three models on the same testing dataset.
(TIF)

S2 Fig Comparison between the balanced model and the Knight’s gestational age model on the placenta testing dataset. (A) Because Knight’s model is a cord blood-based model, it is unsuitable for the placenta data here and shows a much weaker performance than the balanced model. In contrast, the balanced model has an R square of 0.812 and an MSE of 13.0. (B) The balanced model also performs well on sample residuals. (C) The 2 models only share 1 required probe.
(TIF)

S3 Fig The gestational age clocks are associated with pregnancy complication status. (A) and (B) Both the bootstrapped clock (A) and the normal clock (B) show the DNAm gestational ages of normal samples are similar to their chronological one. However, the preeclampsia samples’ DNAm gestational ages are significantly larger than their chronological one, indicating the accelerated senescence.
(TIF)

S4 Fig Performance of the three clock models on the whole blood dataset with probes as features. (A) to (C) Performance of the balanced model (A), bootstrapped model (B), and single normal model (C) on the same training dataset with probes as features. The color gradients of the dots indicate the density of the samples. (D) to (F) Performance of these three models on the same testing dataset with probes as features. (G) and (H) Residuals of the three models in training and testing datasets.
(TIF)

S5 Fig Biological functions of the genes selected from the whole blood dataset. (A) Venn diagram showing the relation among the genes selected by the three models. (B) to (D) Biological function enrichment results for the total genes (B), shared genes (C) of the three models, as well as the genes selected by the single normal model uniquely (D).
(TIF)

S6 Fig Comparison between the balanced model and other 4 lifespan age models on the blood testing dataset. (A) The 3 best models are the BNN model (R square = 0.916, MSE = 20.0), the balanced model (R square = 0.901, MSE = 23.7), and the Horvath’s model (R square = 0.894, MSE = 25.0). (B) The balanced model performs the best on the residuals of the low-density samples with a lifespan age > 40 years old. (C) The models share only a few required probes. Because the BNN model uses Horvath’s probes to train its Bayesian neural network (BNN), the probes of these 2 models are the same.
(TIF)

S7 Fig Performance of the three clock models on the brain dataset with probes as features. (A) to (C) Performance of the balanced model (A), bootstrapped model (B), and single normal model (C) on the same training dataset with probes as features. The color gradients of the dots indicate the density of the samples. (D) to (F) Performance of these three models on the same testing dataset with probes as features. (G) and (H) Residuals of the three models in training and testing datasets.
(TIF)

S8 Fig Biological functions of the genes covered by the DMRs selected from the 450K brain dataset. (A) Venn diagram showing the relation among the DMRs selected by the three models. (B) Venn diagram showing the genes covered by the DMRs selected. (C) and (D) Biological function enrichment results for the total genes (C) and shared genes (D) covered by the DMRs.
(TIF)

S9 Fig Comparison among the balanced model, the bootstrapped model, and other 7 lifespan age models on the brain testing dataset. (A) The 4 best models are the bootstrapped model (R square = 0.918, MSE = 16.4), the Horvath’s model (R square = 0.953, MSE = 24.3), the balanced model (R square = 0.827, MSE = 34.6), and the Hannum’s model (R square = 0.897, MSE = 108). The BNN and PedBE models also had an R square > 0.5, while there are also 3 models with an R square < 0.5 (the Levine’s, Wu’s, and Horvath’s skin models) and are not shown here. (B) The balanced and the bootstrapped models perform the best on the residuals across the samples.
(TIF)

S1 Table Gestational age relevant probes selected by the three models. (XLSX)

S2 Table Lifespan age relevant genes selected from the whole blood dataset by the three models. (XLSX)

S3 Table Lifespan age relevant DMRs selected from the 450K brain dataset by the three models. (XLSX)

S4 Table Overlapping between clock model selected gene features and the GenAge database. (XLSX)

## References

[1] Deaton AM, Bird A. CpG islands and the regulation of transcription. Genes & Development. 2011;25(10):1010–22. doi: 10.1101/gad.2037511 21576262 PMC3093116

[2] Richard MA, Huan T, Ligthart S, Gondalia R, Jhun MA, Brody JA, et al. DNA Methylation Analysis Identifies Loci for Blood Pressure Regulation. The American Journal of Human Genetics. 2017;101(6):888–902. doi: 10.1016/j.ajhg.2017.09.028 29198723 PMC5812919

[3] Wang AL, Gruzieva O, Qiu W, Kebede Merid S, Celedón JC, Raby BA, et al. DNA methylation is associated with inhaled corticosteroid response in persistent childhood asthmatics. Clinical & Experimental Allergy. 2019;49(9):1225–34. doi: 10.1111/cea.13447 31187518 PMC7085934

[4] Yang Z, Wong A, Kuh D, Paul DS, Rakyan VK, Leslie RD, et al. Correlation of an epigenetic mitotic clock with cancer risk. Genome Biology. 2016;17(1):205. doi: 10.1186/s13059-016-1064-3 27716309 PMC5046977

[5] Teschendorff AE, Relton CL. Statistical and integrative system-level analysis of DNA methylation data. Nature Reviews Genetics. 2018;19(3):129–47. doi: 10.1038/nrg.2017.86 29129922

[6] Bell JT, Tsai P-C, Yang T-P, Pidsley R, Nisbet J, Glass D, et al. Epigenome-Wide Scans Identify Differentially Methylated Regions for Age and Age-Related Phenotypes in a Healthy Ageing Population. PLOS Genetics. 2012;8(4):e1002629. doi: 10.1371/journal.pgen.1002629 22532803 PMC3330116

[7] Horvath S, Zhang Y, Langfelder P, Kahn RS, Boks MPM, van Eijk K, et al. Aging effects on DNA methylation modules in human brain and blood tissue. Genome Biology. 2012;13(10):R97. doi: 10.1186/gb-2012-13-10-r97 23034122 PMC4053733

[8] Horvath S. DNA methylation age of human tissues and cell types. Genome Biology. 2013;14(10):3156. doi: 10.1186/gb-2013-14-10-r115 24138928 PMC4015143

[9] Hannum G, Guinney J, Zhao L, Zhang L, Hughes G, Sadda S, et al. Genome-wide methylation profiles reveal quantitative views of human aging rates. Mol Cell. 2013;49(2):359–67. Epub 2012/11/28. doi: 10.1016/j.molcel.2012.10.016 ; PubMed Central PMCID: PMC3780611. 23177740 PMC3780611

[10] Bocklandt S, Lin W, Sehl ME, Sánchez FJ, Sinsheimer JS, Horvath S, et al. Epigenetic Predictor of Age. PLOS ONE. 2011;6(6):e14821. doi: 10.1371/journal.pone.0014821 21731603 PMC3120753

[11] Weidner CI, Lin Q, Koch CM, Eisele L, Beier F, Ziegler P, et al. Aging of blood can be tracked by DNA methylation changes at just three CpG sites. Genome Biology. 2014;15(2):R24. doi: 10.1186/gb-2014-15-2-r24 24490752 PMC4053864

[12] Ambatipudi S, Horvath S, Perrier F, Cuenin C, Hernandez-Vargas H, Le Calvez-Kelm F, et al. DNA methylome analysis identifies accelerated epigenetic ageing associated with postmenopausal breast cancer susceptibility. European Journal of Cancer. 2017;75:299–307. doi: 10.1016/j.ejca.2017.01.014 28259012 PMC5512160

[13] Levine ME, Hosgood HD, Chen B, Absher D, Assimes T, Horvath S. DNA methylation age of blood predicts future onset of lung cancer in the women’s health initiative. Aging. 2015;7(9):690–700. doi: 10.18632/aging.100809 26411804 PMC4600626

[14] Roetker Nicholas S, Pankow James S, Bressler J, Morrison Alanna C, Boerwinkle E. Prospective Study of Epigenetic Age Acceleration and Incidence of Cardiovascular Disease Outcomes in the ARIC Study (Atherosclerosis Risk in Communities). Circulation: Genomic and Precision Medicine. 2018;11(3):e001937. doi: 10.1161/CIRCGEN.117.001937 29555670 PMC5863591

[15] Ferrucci L, Gonzalez-Freire M, Fabbri E, Simonsick E, Tanaka T, Moore Z, et al. Measuring biological aging in humans: A quest. Aging Cell. 2020;19(2):e13080. doi: 10.1111/acel.13080 31833194 PMC6996955

[16] Mayne BT, Leemaqz SY, Smith AK, Breen J, Roberts CT, Bianco-Miotto T. Accelerated placental aging in early onset preeclampsia pregnancies identified by DNA methylation. Epigenomics. 2016;9(3):279–89. doi: 10.2217/epi-2016-0103 27894195 PMC6040051

[17] Lee Y, Choufani S, Weksberg R, Wilson SL, Yuan V, Burt A, et al. Placental epigenetic clocks: estimating gestational age using placental DNA methylation levels. Aging. 2019;11(12):4238–53. doi: 10.18632/aging.102049 31235674 PMC6628997

[18] Young PC, Glasgow TS, Li X, Guest-Warnick G, Stoddard G. Mortality of Late-Preterm (Near-Term) Newborns in Utah. Pediatrics. 2007;119(3):e659. doi: 10.1542/peds.2006-2486 17332185

[19] Engle WA. Morbidity and Mortality in Late Preterm and Early Term Newborns: A Continuum. Clinics in Perinatology. 2011;38(3):493–516. doi: 10.1016/j.clp.2011.06.009 21890021

[20] Yang S, Platt RW, Kramer MS. Variation in Child Cognitive Ability by Week of Gestation Among Healthy Term Births. American Journal of Epidemiology. 2010;171(4):399–406. doi: 10.1093/aje/kwp413 20080810 PMC3435092

[21] Davis E, Buss C, Muftuler T, Head K, Hasso A, Wing D, et al. Children’s Brain Development Benefits from Longer Gestation. Frontiers in Psychology. 2011;2:1. doi: 10.3389/fpsyg.2011.00001 21713130 PMC3111445

[22] Hansen AK, Wisborg K, Uldbjerg N, Henriksen TB. Risk of respiratory morbidity in term infants delivered by elective caesarean section: cohort study. BMJ. 2008;336(7635):85. doi: 10.1136/bmj.39405.539282.BE 18077440 PMC2190264

[23] Parikh LI, Reddy UM, Männistö T, Mendola P, Sjaarda L, Hinkle S, et al. Neonatal outcomes in early term birth. American Journal of Obstetrics & Gynecology. 2014;211(3):265.e1–.e11. doi: 10.1016/j.ajog.2014.03.021 24631438 PMC4149822

[24] Ding Z, Zu S, Gu J. Evaluating the molecule-based prediction of clinical drug responses in cancer. Bioinformatics (Oxford, England). 2016;32(19):2891–5. Epub 2016/06/30. doi: 10.1093/bioinformatics/btw344 . 27354694

[25] Novakovic B, Yuen RK, Gordon L, Penaherrera MS, Sharkey A, Moffett A, et al. Evidence for widespread changes in promoter methylation profile in human placenta in response to increasing gestational age and environmental/stochastic factors. BMC Genomics. 2011;12(1):529. doi: 10.1186/1471-2164-12-529 22032438 PMC3216976

[26] Chu T, Bunce K, Shaw P, Shridhar V, Althouse A, Hubel C, et al. Comprehensive Analysis of Preeclampsia-Associated DNA Methylation in the Placenta. PLOS ONE. 2014;9(9):e107318. doi: 10.1371/journal.pone.0107318 25247495 PMC4172433

[27] Hanna CW, Peñaherrera MS, Saadeh H, Andrews S, McFadden DE, Kelsey G, et al. Pervasive polymorphic imprinted methylation in the human placenta. Genome Res. 2016;26(6):756–67. Epub 2016/01/16. doi: 10.1101/gr.196139.115 ; PubMed Central PMCID: PMC4889973. 26769960 PMC4889973

[28] Price EM, Peñaherrera MS, Portales-Casamar E, Pavlidis P, Van Allen MI, McFadden DE, et al. Profiling placental and fetal DNA methylation in human neural tube defects. Epigenetics & Chromatin. 2016;9(1):6. doi: 10.1186/s13072-016-0054-8 26889207 PMC4756451

[29] Leavey K, Wilson SL, Bainbridge SA, Robinson WP, Cox BJ. Epigenetic regulation of placental gene expression in transcriptional subtypes of preeclampsia. Clinical Epigenetics. 2018;10(1):28. doi: 10.1186/s13148-018-0463-6 29507646 PMC5833042

[30] Wilson SL, Leavey K, Cox BJ, Robinson WP. Mining DNA methylation alterations towards a classification of placental pathologies. Hum Mol Genet. 2018;27(1):135–46. Epub 2017/11/02. doi: 10.1093/hmg/ddx391 ; PubMed Central PMCID: PMC5886226. 29092053 PMC5886226

[31] Zhou W, Triche TJ Jr., Laird PW, Shen H. SeSAMe: reducing artifactual detection of DNA methylation by Infinium BeadChips in genomic deletions. Nucleic acids research. 2018;46(20):e123–e. doi: 10.1093/nar/gky691 30085201 PMC6237738

[32] Triche TJ Jr., Weisenberger DJ, Van Den Berg D, Laird PW, Siegmund KD. Low-level processing of Illumina Infinium DNA Methylation BeadArrays. Nucleic acids research. 2013;41(7):e90–e. doi: 10.1093/nar/gkt090 23476028 PMC3627582

[33] Guintivano J, Aryee MJ, Kaminsky ZA. A cell epigenotype specific model for the correction of brain cellular heterogeneity bias and its application to age, brain region and major depression. Epigenetics. 2013;8(3):290–302. doi: 10.4161/epi.23924 23426267 PMC3669121

[34] Friedman J, Hastie T, Tibshirani R. Regularization Paths for Generalized Linear Models via Coordinate Descent. J Stat Softw. 2010;33(1):1–22. Epub 2010/09/03. ; PubMed Central PMCID: PMC2929880. 20808728 PMC2929880

[35] Simon N, Friedman J, Hastie T, Tibshirani R. Regularization Paths for Cox’s Proportional Hazards Model via Coordinate Descent. Journal of statistical software. 2011;39(5):1–13. Epub 2011/03/01. doi: 10.18637/jss.v039.i05 ; PubMed Central PMCID: PMC4824408. 27065756 PMC4824408

[36] Tibshirani R, Bien J, Friedman J, Hastie T, Simon N, Taylor J, et al. Strong rules for discarding predictors in lasso-type problems. J R Stat Soc Series B Stat Methodol. 2012;74(2):245–66. Epub 2012/03/01. doi: 10.1111/j.1467-9868.2011.01004.x ; PubMed Central PMCID: PMC4262615. 25506256 PMC4262615

[37] Chen EY, Tan CM, Kou Y, Duan Q, Wang Z, Meirelles GV, et al. Enrichr: interactive and collaborative HTML5 gene list enrichment analysis tool. BMC Bioinformatics. 2013;14(1):128. doi: 10.1186/1471-2105-14-128 23586463 PMC3637064

[38] Kuleshov MV, Jones MR, Rouillard AD, Fernandez NF, Duan Q, Wang Z, et al. Enrichr: a comprehensive gene set enrichment analysis web server 2016 update. Nucleic acids research. 2016;44(W1):W90–7. Epub 2016/05/05. doi: 10.1093/nar/gkw377 ; PubMed Central PMCID: PMC4987924. 27141961 PMC4987924

[39] Pelegí-Sisó D, de Prado P, Ronkainen J, Bustamante M, González JR. methylclock: a Bioconductor package to estimate DNA methylation age. Bioinformatics. 2021;37(12):1759–60. Epub 2020/09/23. doi: 10.1093/bioinformatics/btaa825 . 32960939

[40] Knight AK, Craig JM, Theda C, Bækvad-Hansen M, Bybjerg-Grauholm J, Hansen CS, et al. An epigenetic clock for gestational age at birth based on blood methylation data. Genome Biol. 2016;17(1):206. Epub 2016/10/09. doi: 10.1186/s13059-016-1068-z ; PubMed Central PMCID: PMC5054584. 27717399 PMC5054584

[41] Salama R, Sadaie M, Hoare M, Narita M. Cellular senescence and its effector programs. Genes & Development. 2014;28(2):99–114. doi: 10.1101/gad.235184.113 24449267 PMC3909793

[42] Tacutu R, Thornton D, Johnson E, Budovsky A, Barardo D, Craig T, et al. Human Ageing Genomic Resources: new and updated databases. Nucleic Acids Res. 2018;46(D1):D1083–d90. Epub 2017/11/10. doi: 10.1093/nar/gkx1042 ; PubMed Central PMCID: PMC5753192. 29121237 PMC5753192

[43] Alfonso G, Gonzalez JR. Bayesian neural networks for the optimisation of biological clocks in humans. bioRxiv. 2020:2020.04.21.052605. doi: 10.1101/2020.04.21.052605

[44] Levine ME, Lu AT, Quach A, Chen BH, Assimes TL, Bandinelli S, et al. An epigenetic biomarker of aging for lifespan and healthspan. Aging (Albany NY). 2018;10(4):573–91. Epub 2018/04/21. doi: 10.18632/aging.101414 ; PubMed Central PMCID: PMC5940111. 29676998 PMC5940111

[45] Wu X, Chen W, Lin F, Huang Q, Zhong J, Gao H, et al. DNA methylation profile is a quantitative measure of biological aging in children. Aging (Albany NY). 2019;11(22):10031–51. Epub 2019/11/23. doi: 10.18632/aging.102399 ; PubMed Central PMCID: PMC6914436. 31756171 PMC6914436

[46] McEwen LM, O’Donnell KJ, McGill MG, Edgar RD, Jones MJ, MacIsaac JL, et al. The PedBE clock accurately estimates DNA methylation age in pediatric buccal cells. Proc Natl Acad Sci U S A. 2020;117(38):23329–35. Epub 2019/10/16. doi: 10.1073/pnas.1820843116 ; PubMed Central PMCID: PMC7519312. 31611402 PMC7519312

[47] Vanyushin BF, Nemirovsky LE, Klimenko VV, Vasiliev VK, Belozersky AN. The 5-methylcytosine in DNA of rats. Tissue and age specificity and the changes induced by hydrocortisone and other agents. Gerontologia. 1973;19(3):138–52. Epub 1973/01/01. . 4763637

[48] Wilson VL, Smith RA, Ma S, Cutler RG. Genomic 5-methyldeoxycytidine decreases with age. The Journal of biological chemistry. 1987;262(21):9948–51. Epub 1987/07/25. . 3611071

