# Supplementary figures and images for: eClock: An ensemble-based method to accurately predict ages with a biased distribution from DNA methylation data
Source: PLoS One. 2022 May 6;17(5):e0267349. doi: 10.1371/journal.pone.0267349 (PMC9075636; doi:10.1371/journal.pone.0267349)

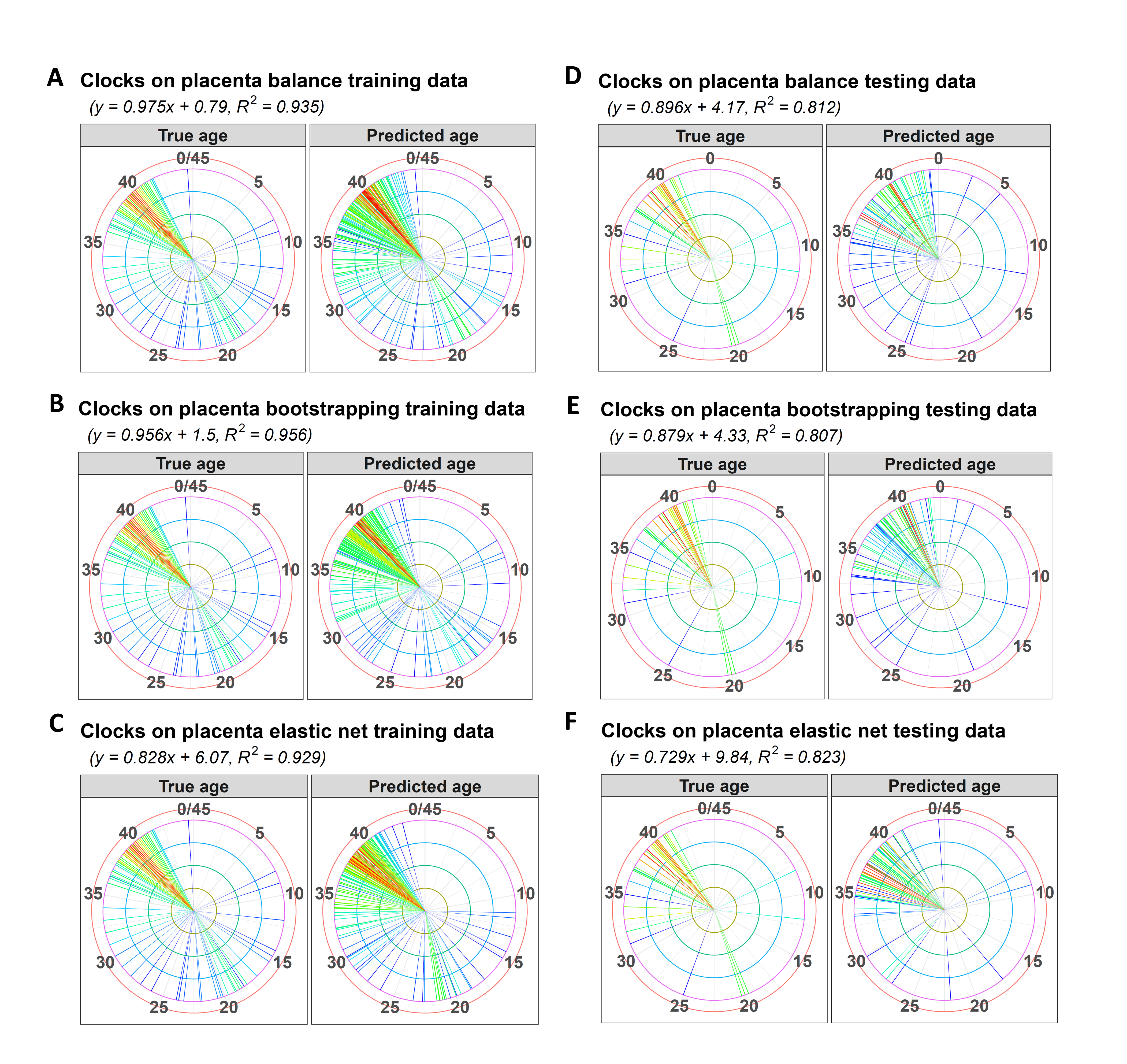

Supplement: S1 Fig — (A) to (C) Clock plots generated by our package show the clock model performance on the same training dataset. The scale around the dial indicates the gestational age, and each pointer represents one sample. The color gradients of the pointers indicate the density of the samples. (A) is the result for the balanced model, (B) is for the bootstrapped model, and (C) is for the single normal model. (D) to (F) Performance of these three models on the same testing dataset. (TIF) [file pone.0267349.s001.tif]

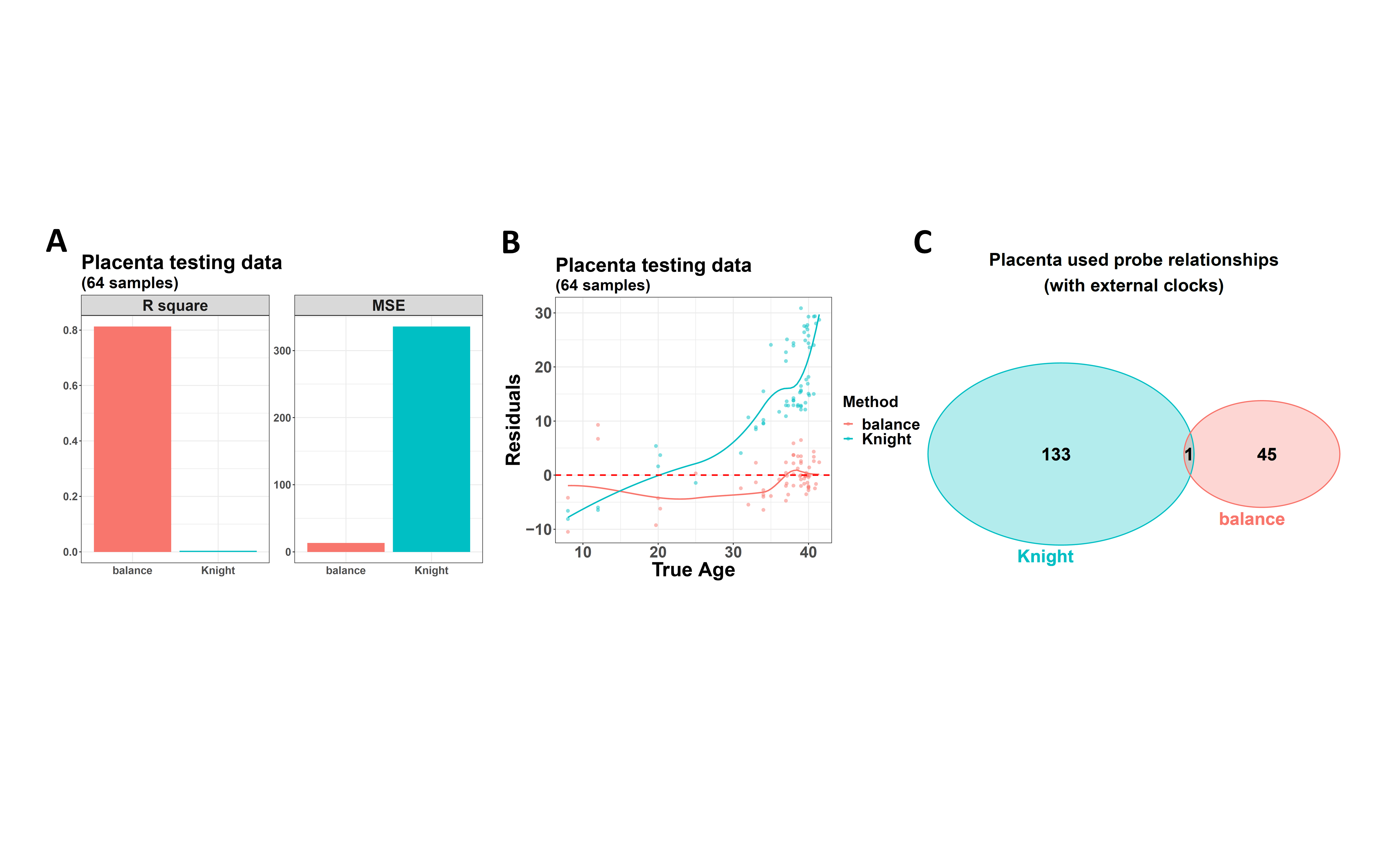

Supplement: S2 Fig — (A) Because Knight’s model is a cord blood-based model, it is unsuitable for the placenta data here and shows a much weaker performance than the balanced model. In contrast, the balanced model has an R square of 0.812 and an MSE of 13.0. (B) The balanced model also performs well on sample residuals. (C) The 2 models only share 1 required probe. (TIF) [file pone.0267349.s002.tif]

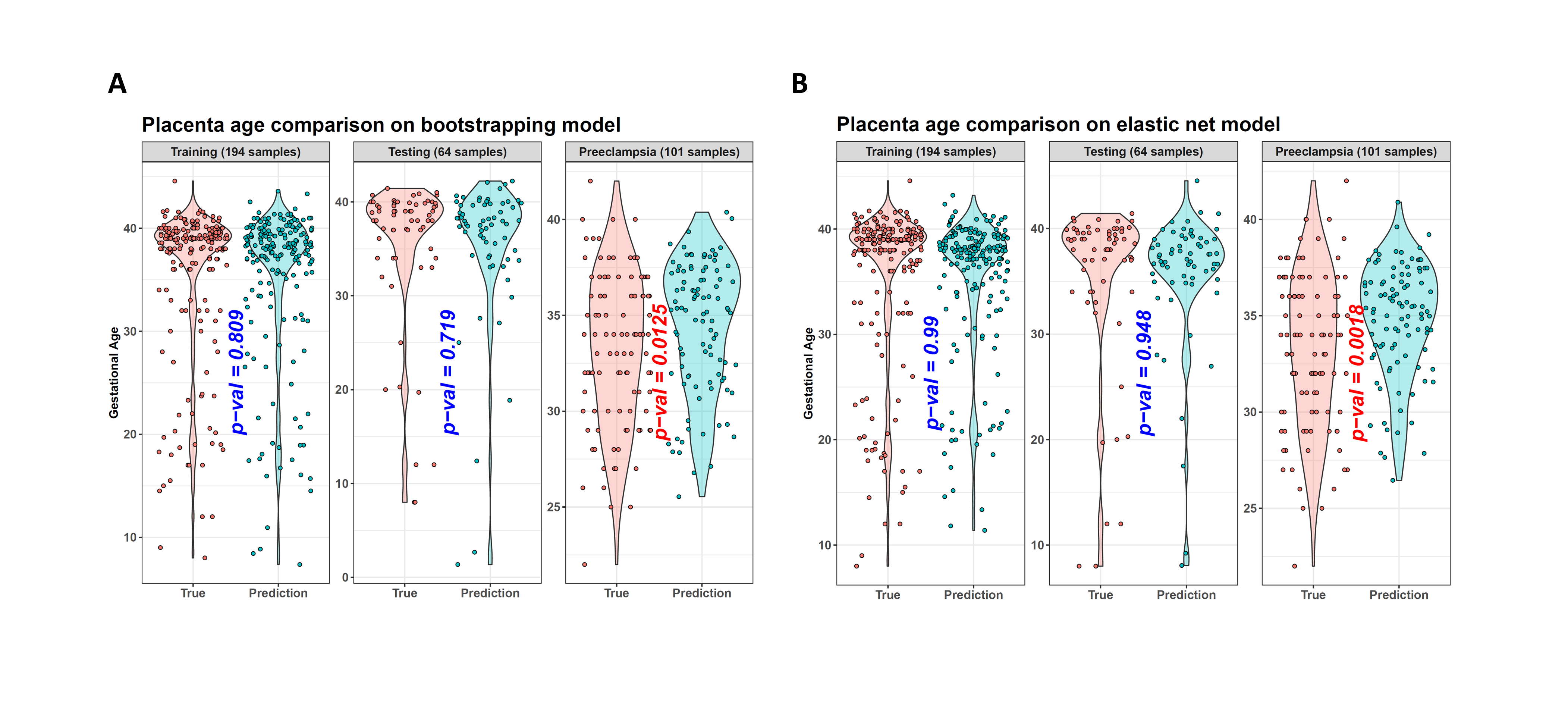

Supplement: S3 Fig — (A) and (B) Both the bootstrapped clock (A) and the normal clock (B) show the DNAm gestational ages of normal samples are similar to their chronological one. However, the preeclampsia samples’ DNAm gestational ages are significantly larger than their chronological one, indicating the accelerated senescence. (TIF) [file pone.0267349.s003.tif]

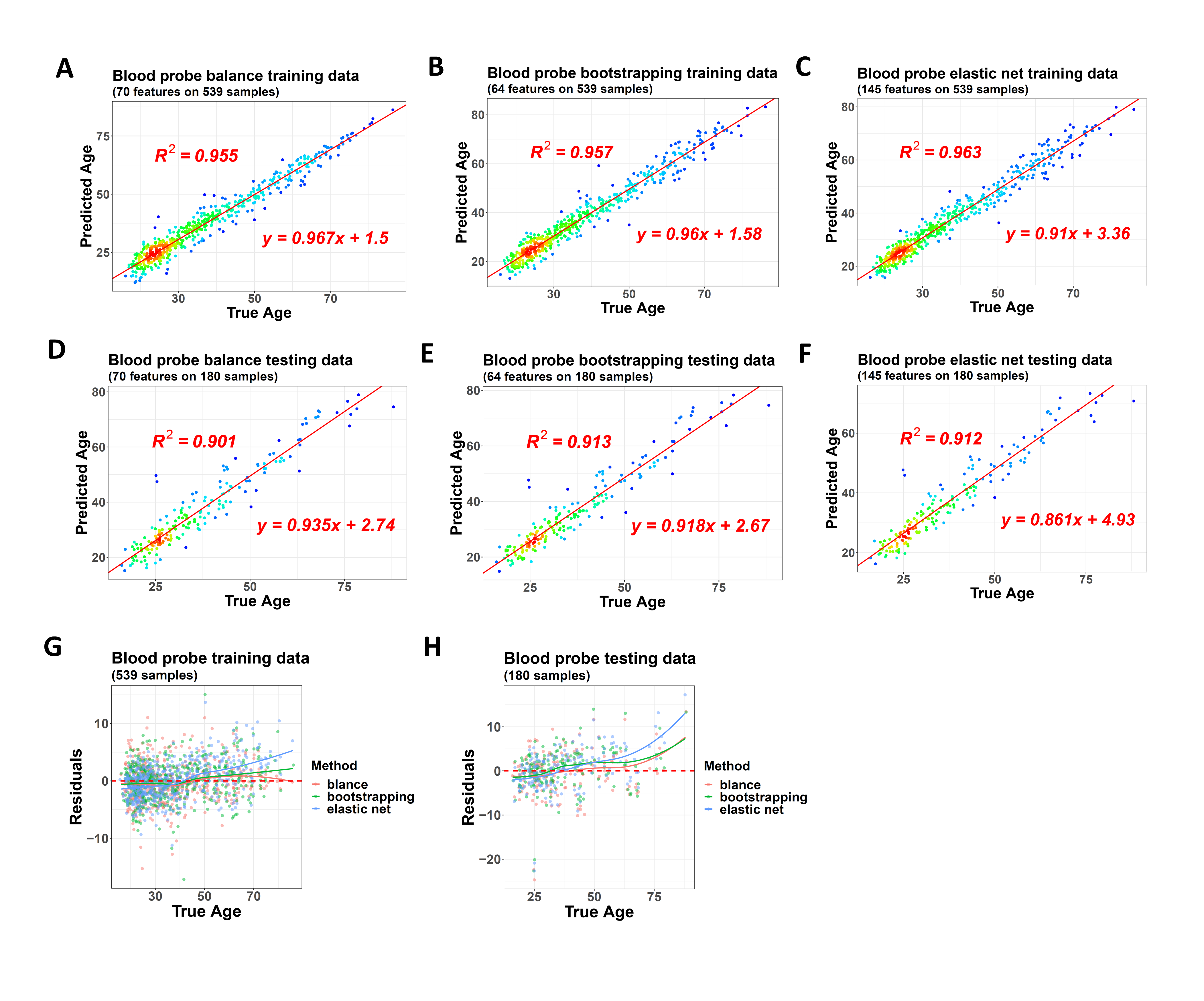

Supplement: S4 Fig — (A) to (C) Performance of the balanced model (A), bootstrapped model (B), and single normal model (C) on the same training dataset with probes as features. The color gradients of the dots indicate the density of the samples. (D) to (F) Performance of these three models on the same testing dataset with probes as features. (G) and (H) Residuals of the three models in training and testing datasets. (TIF) [file pone.0267349.s004.tif]

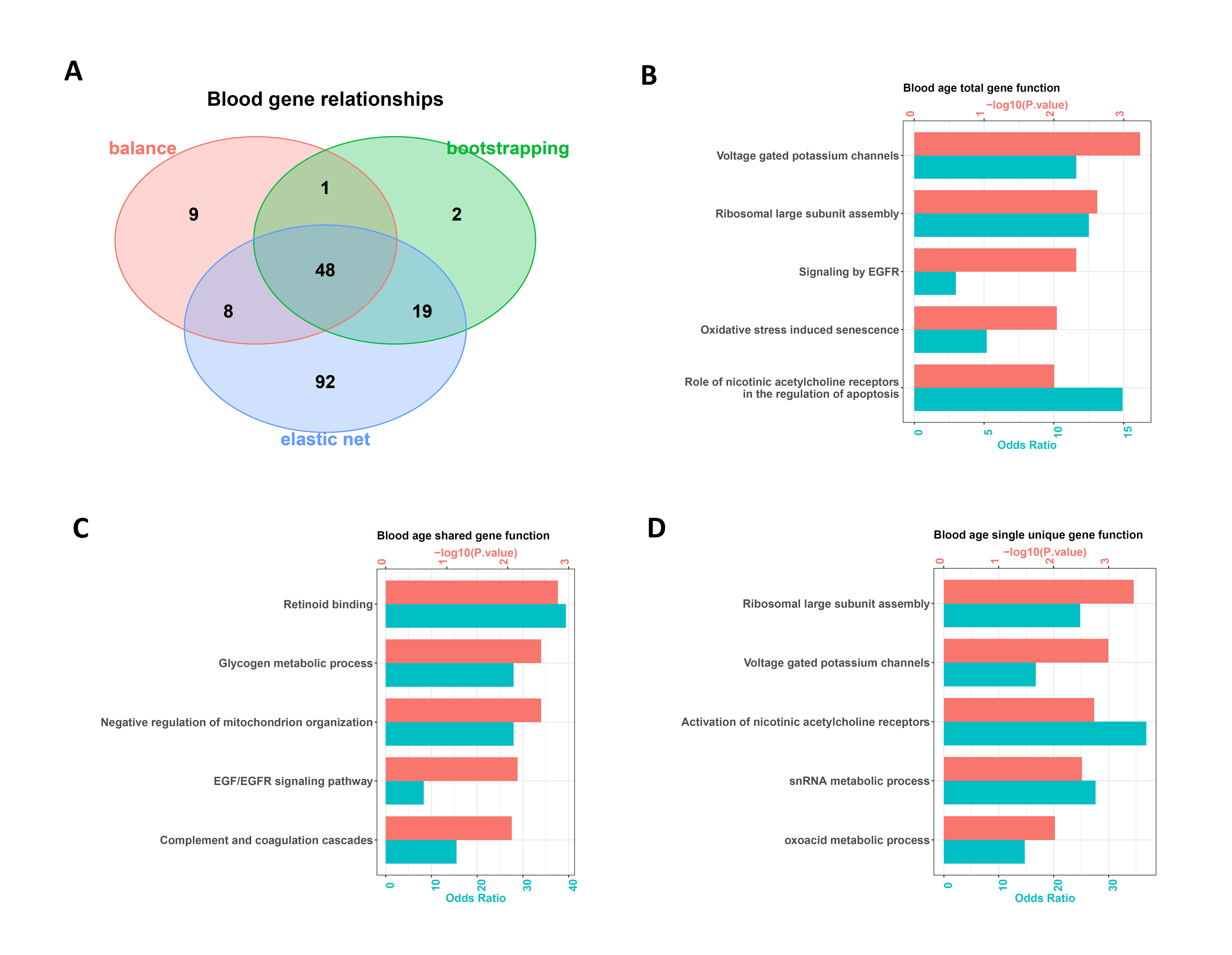

Supplement: S5 Fig — (A) Venn diagram showing the relation among the genes selected by the three models. (B) to (D) Biological function enrichment results for the total genes (B), shared genes (C) of the three models, as well as the genes selected by the single normal model uniquely (D). (TIF) [file pone.0267349.s005.tif]

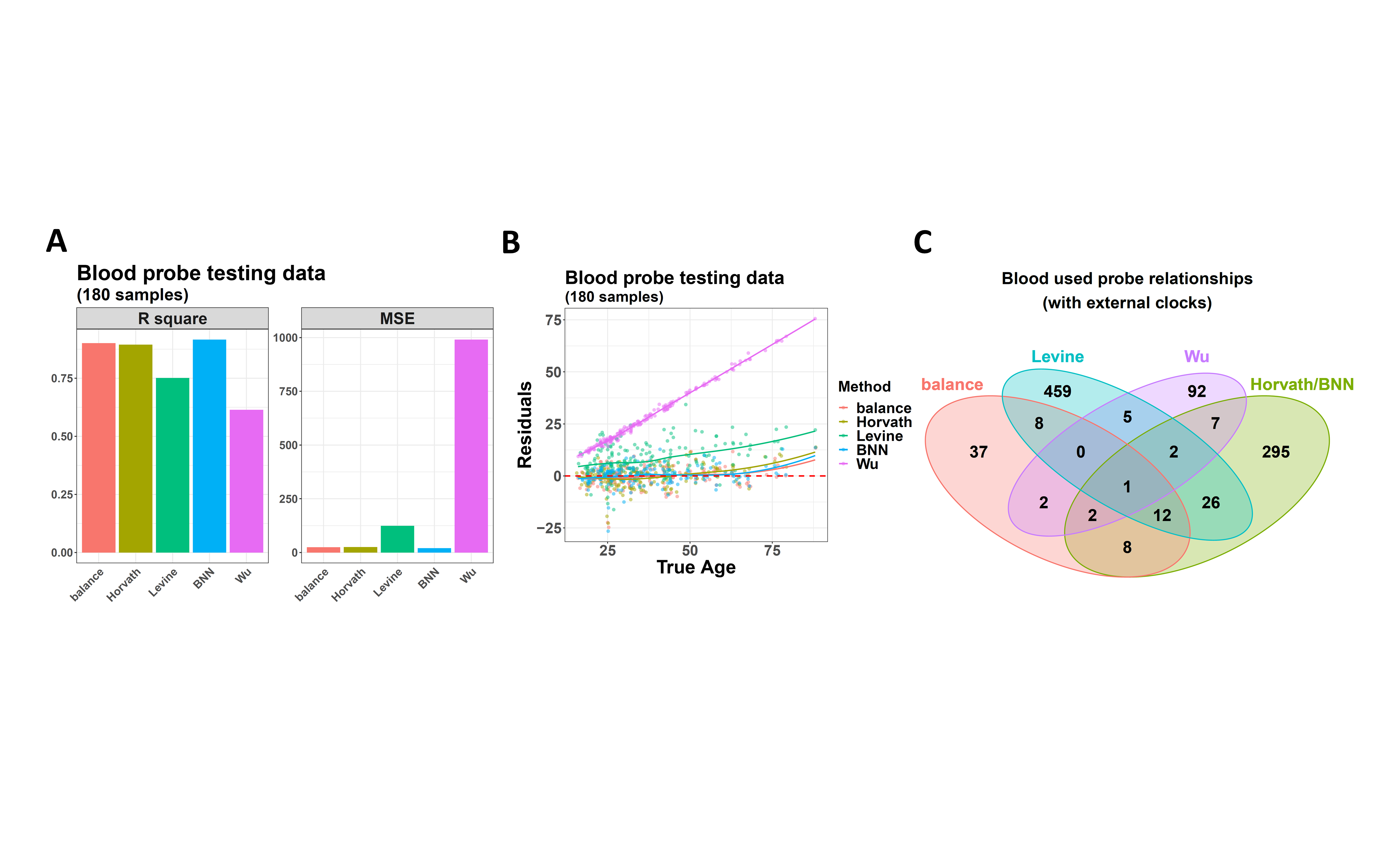

Supplement: S6 Fig — (A) The 3 best models are the BNN model (R square = 0.916, MSE = 20.0), the balanced model (R square = 0.901, MSE = 23.7), and the Horvath’s model (R square = 0.894, MSE = 25.0). (B) The balanced model performs the best on the residuals of the low-density samples with a lifespan age > 40 years old. (C) The models share only a few required probes. Because the BNN model uses Horvath’s probes to train its Bayesian neural network (BNN), the probes of these 2 models are the same. (TIF) [file pone.0267349.s006.tif]

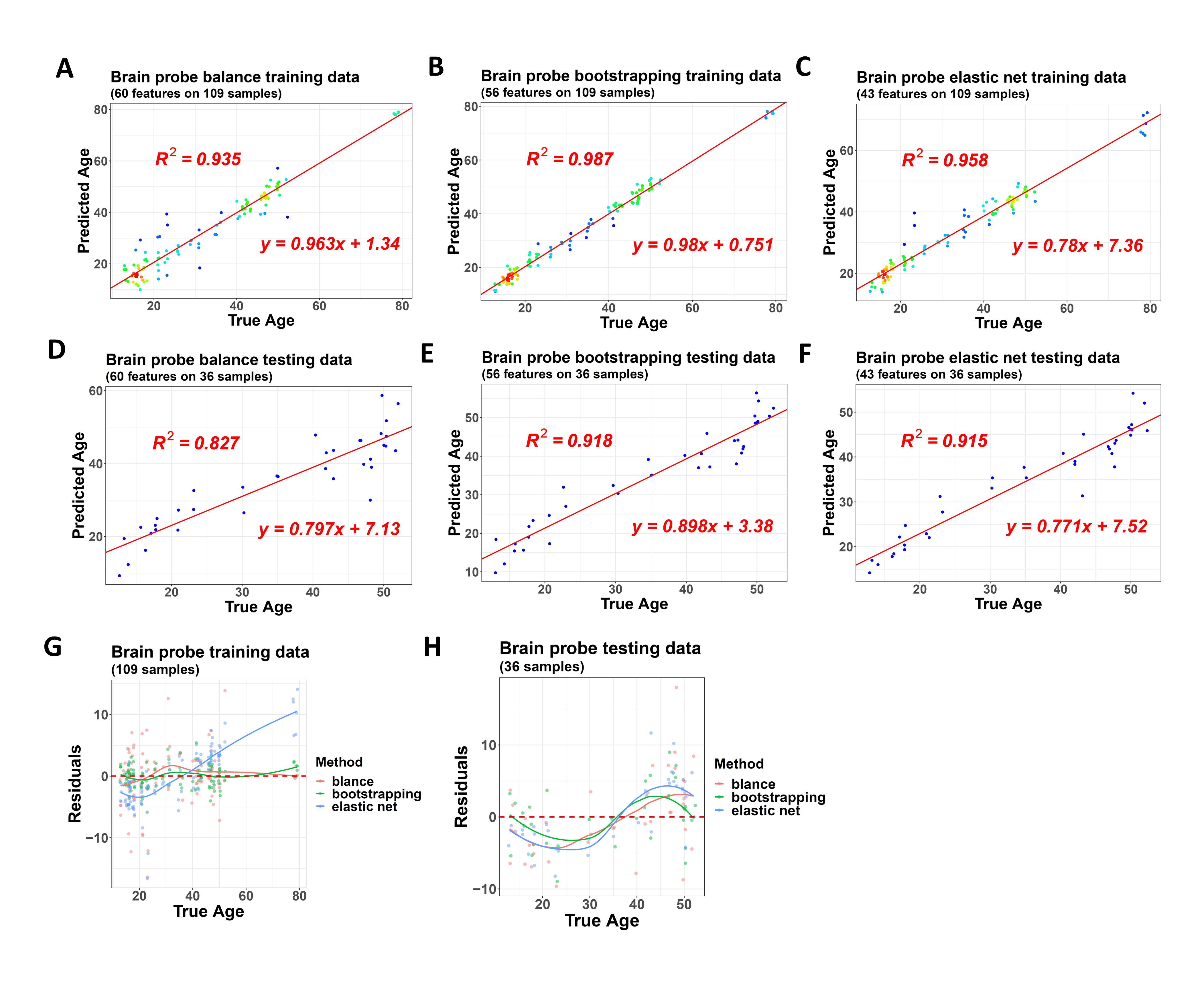

Supplement: S7 Fig — (A) to (C) Performance of the balanced model (A), bootstrapped model (B), and single normal model (C) on the same training dataset with probes as features. The color gradients of the dots indicate the density of the samples. (D) to (F) Performance of these three models on the same testing dataset with probes as features. (G) and (H) Residuals of the three models in training and testing datasets. (TIF) [file pone.0267349.s007.tif]

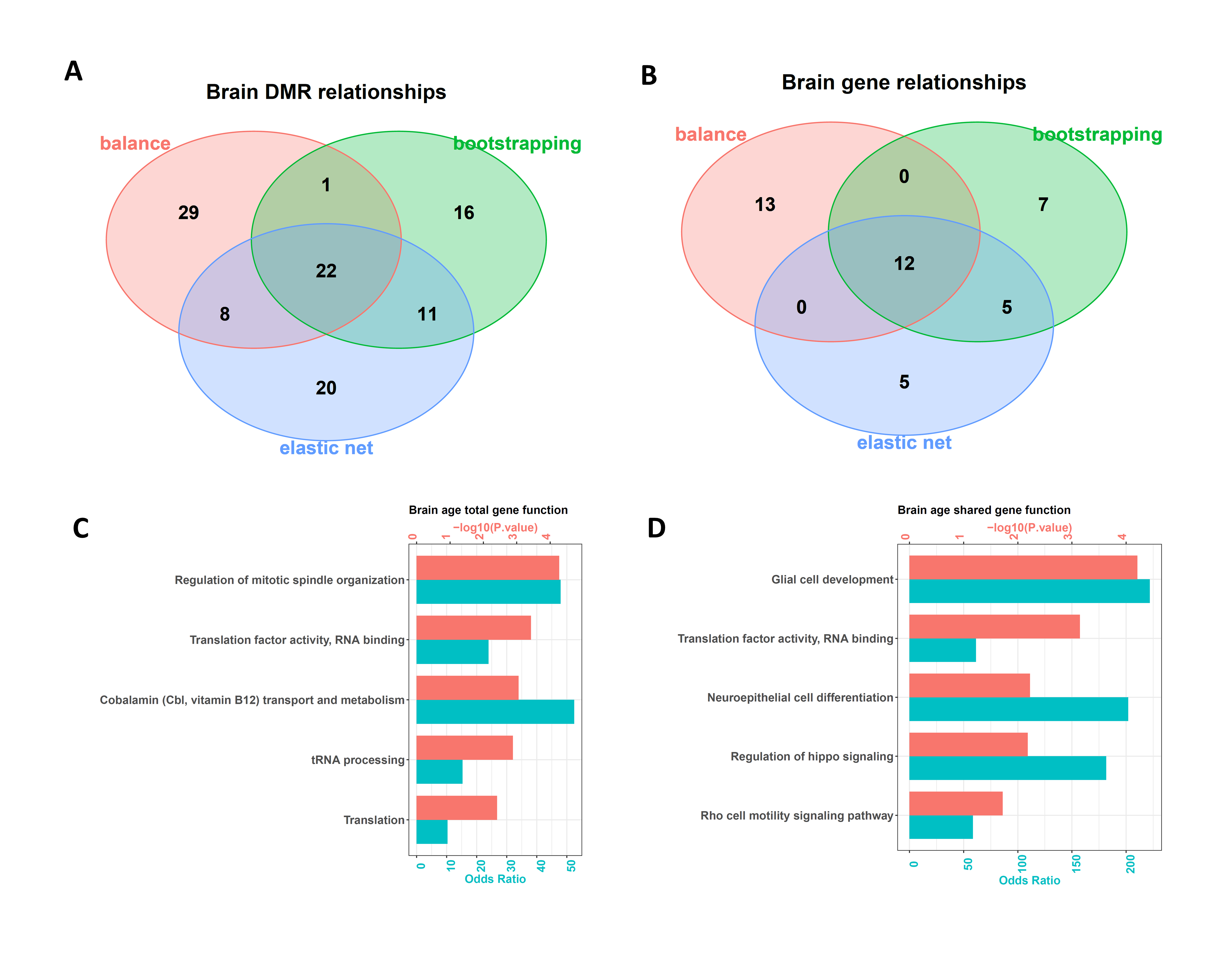

Supplement: S8 Fig — (A) Venn diagram showing the relation among the DMRs selected by the three models. (B) Venn diagram showing the genes covered by the DMRs selected. (C) and (D) Biological function enrichment results for the total genes (C) and shared genes (D) covered by the DMRs. (TIF) [file pone.0267349.s008.tif]

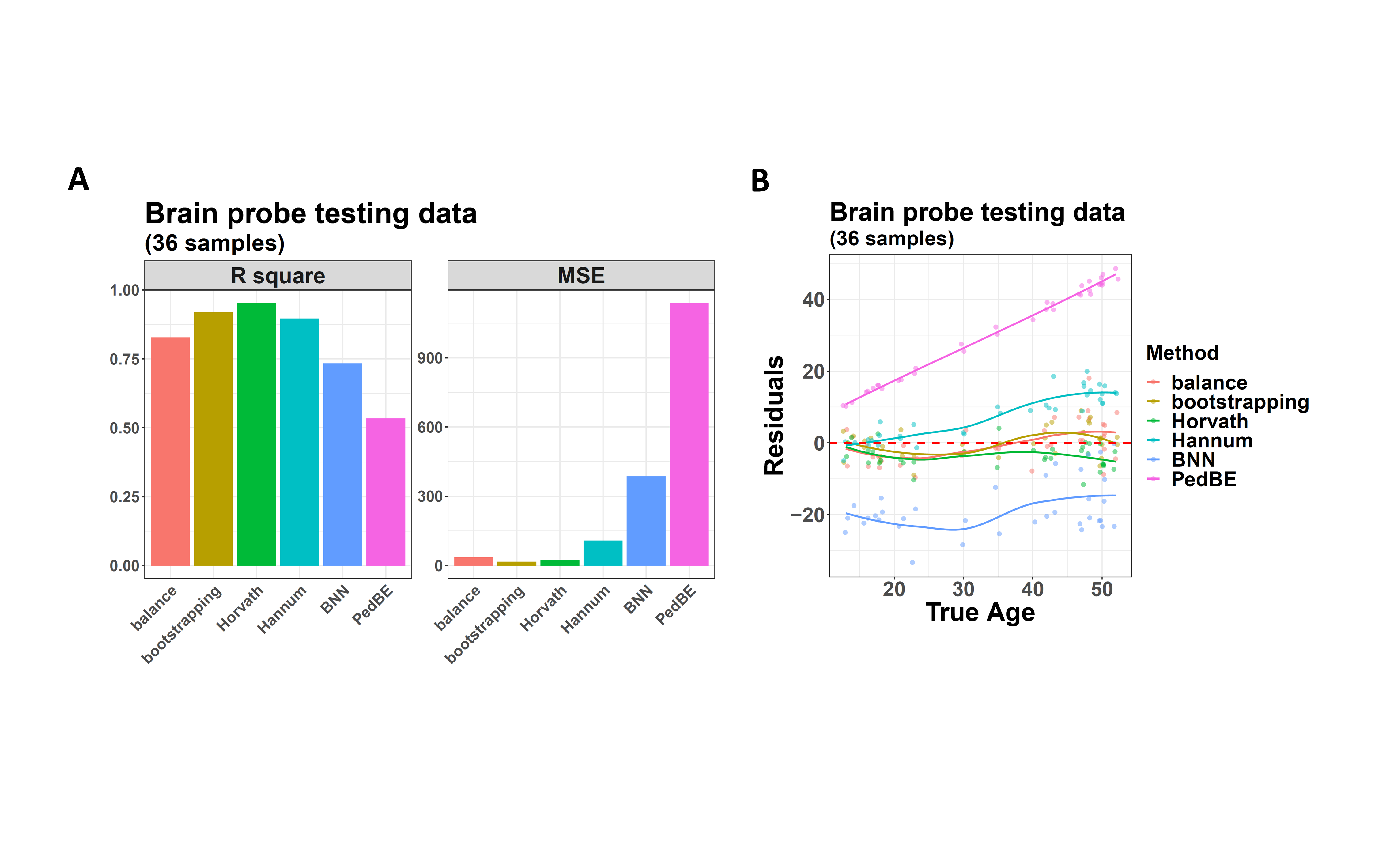

Supplement: S9 Fig — (A) The 4 best models are the bootstrapped model (R square = 0.918, MSE = 16.4), the Horvath’s model (R square = 0.953, MSE = 24.3), the balanced model (R square = 0.827, MSE = 34.6), and the Hannum’s model (R square = 0.897, MSE = 108). The BNN and PedBE models also had an R square > 0.5, while there are also 3 models with an R square < 0.5 (the Levine’s, Wu’s, and Horvath’s skin models) and are not shown here. (B) The balanced and the bootstrapped models perform the best on the residuals across the samples. (TIF) [file pone.0267349.s009.tif]
